# Structural and functional mapping of protective human monoclonal antibodies against enterovirus A71

**DOI:** 10.1126/sciadv.aee8217

**Published:** 2026-06-05

**Authors:** Daming Zhou, Abhay Kotecha, James T. Kelly, Peng-Nien Huang, Yi-Yin Chen, Thomas S. Walter, Helen M.E. Duyvesteyn, Raymond J. Owens, Shu-Yuan Ho, Tzou-Yien Lin, Elizabeth E. Fry, Jingshan Ren, Kuan-Ying A. Huang, David I. Stuart

**Affiliations:** ^1^ College of Life Sciences, Zhejiang University, Hangzhou, China.; ^2^ Chinese Academy of Medical Science (CAMS) Oxford Institute (COI), University of Oxford, Oxford, UK.; ^3^ Division of Structural Biology, Centre for Human Genetics, Nuffield Department of Medicine, University of Oxford, Oxford, UK.; ^4^ St. Jude Children’s Research Hospital, Inspiration 4 Advanced Research Center, 262 Danny Thomas Place, Memphis, TN, USA.; ^5^ The Pirbright Institute, Woking, Surrey, UK.; ^6^ Research Center for Emerging Viral Infections, College of Medicine, Chang Gung University, Taoyuan, Taiwan.; ^7^ Division of Pediatric Infectious Diseases, Department of Pediatrics, Linkou Chang Gung Memorial Hospital, Taoyuan, Taiwan.; ^8^ Graduate Institute of Immunology and Department of Pediatrics, National Taiwan University Hospital, College of Medicine, National Taiwan University, Taipei, Taiwan.; ^9^ The Rosalind Franklin Institute, Harwell Campus, Didcot, UK.; ^10^ Department of Laboratory Medicine, National Taiwan University Hospital and National Taiwan University College of Medicine, Taipei, Taiwan.; ^11^ Division of Pediatric Infectious Diseases, Department of Pediatrics, Chang Gung Memorial Hospital, Taoyuan, Taiwan.; ^12^ College of Medicine, Chang Gung University, Taoyuan, Taiwan.; ^13^ Genomics Research Center, Academia Sinica, Taipei, Taiwan.

## Abstract

EV-A71 has been responsible for recent severe HFMD outbreaks. We report structures for 12 potently neutralizing human anti–EV-A71 monoclonal antibody Fabs, alone and complexed with virus. Most recognize the native antigenic state with epitopes that span interfaces, together covering 85% of the capsid surface. The majority (8 of 12) bind the canyon, while the others cluster around the icosahedral two- and threefold axes. Blocking SCARB2 receptor binding likely contributes to neutralization for all, and a subset induces empty particles. A predominant gene family (IGHV4-39) does not dictate a common binding pose. Long CDR-H3 loops are frequently key to binding, especially at the canyon, suggesting that antigenicity data based on antibodies with shorter CDR3s (e.g., murine) may be misleading. This dataset reveals neutralization mechanisms for recently circulating EV-A71 genotypes, which will inform immunotherapies. We demonstrate synergy in vitro between canyon binding and both two- and threefold binding antibodies to increase neutralization potency.

## Teaser

Human antibodies bind across most of the EV-A71 capsid, often neutralizing the virus by reaching deep into a crevice.

## INTRODUCTION

Hand-foot-and-mouth disease (HFMD) primarily affects infants and young children, presenting with fever, vesicular eruptions on the hands and feet, and oral lesions. Although globally distributed, the most severe outbreaks have been reported in the Asia-Pacific region over the past two decades (*1*). According to World Health Organization (WHO) reports, ∼2,000,000 cases occur annually in Asian countries, with enterovirus 71 (EV-A71) and coxsackievirus A16 (CV-A16) as the principal causative agents (*2*, *3*). Among these, EV-A71 is responsible for the most serious outcomes, including severe neurological and cardiac complications that can result in fatalities (*3*).

EV-A71 is a positive-sense, single-stranded RNA virus of the genus *Enterovirus* (family Picornaviridae). On the basis of the VP1 gene sequence, it is classified into three genotypes (A, B, and C), which are further subdivided into multiple subtypes (B1 to B5, C1 to C5) and strains (*4*). The virus is nonenveloped, with an ∼30-nm icosahedral capsid composed of 60 copies each of VP1 to VP4. VP1 to VP3 form the outer surface, while VP4 lines the interior, which harbors the RNA genome. A prominent surface feature is the canyon encircling each fivefold axis; beneath the canyon floor in VP1 lies a hydrophobic pocket that accommodates a host-derived lipid pocket factor, stabilizing the native structure of the capsid (*5*, *6*).

Infection is initiated by interaction of the EV-A71 capsid with host receptors, followed by internalization and uncoating. Several receptors have been implicated, but SCARB2 (scavenger receptor class B member 2) is recognized as the primary receptor: It mediates cell attachment and is essential for pH-dependent uncoating (*7*). We previously reported the structure of EV-A71 bound to SCARB2, demonstrating that this relatively bulky receptor engages the southern rim of the canyon (*8*). Following receptor binding and uptake, acidification drives transitions from the native 160S (N antigenic form) to the expanded 135S (H antigenic form) and ultimately to the empty 80S particle following genome release (*5*).

Monoclonal antibodies (mAbs) represent potential therapeutics against EV-A71. Most described anti–EV-A71 mAbs have been derived from immunized mice, showing efficacy in vitro and in murine challenge models (*9*–*12*). Human-derived antibodies are more suitable for clinical application due to their reduced immunogenicity and appropriate pharmacokinetics; however, relatively few have been reported for EV-A71. Notably, two potent human antibodies, 38-1-10A [Protein Data Bank (PDB) ID: 6Z3K] and 38-3-11A (PDB ID: 6Z3P), were shown to bind the threefold axis region of the EV-A71 capsid (*13*), but it is expected that other human antibodies target distinct epitopes.

We recently identified and characterized 12 human neutralizing mAbs from pediatric cases of EV-A71 infection (*14*). Antibody 34-1-6D was isolated from donor 34 (3 years old); antibodies 17-1-12A, 17-2-2B, and 17-2-12A were isolated from donor 17 (6 years old); and the remaining eight were isolated from donor 16 (4 years old). All three children were infected with closely related strains of EV-A71 genotype B5 and hospitalized with HFMD (*14*). These antibodies displayed variable potency and breadth of neutralization across EV-A71 strains, being generally more effective against recent B5 and C4 isolates than earlier C1 and C2 genotypes (table S1). Escape mutations selected in vitro localized to the canyon region or the plateau regions near the twofold and threefold symmetry axes on the viral capsid (*14*). Although these antibodies hold promise as therapeutic candidates, their neutralization mechanisms have remained unclear. Here, we report the crystal structures of 12 Fabs determined by x-ray crystallography, together with cryo–electron microscopy (cryo-EM) structures of their complexes with EV-A71, providing detailed insights into their epitopes and mechanisms of neutralization.

## RESULTS

### Antibodies broadly neutralize recently circulating EV-A71 viruses

**Fig. 1.**
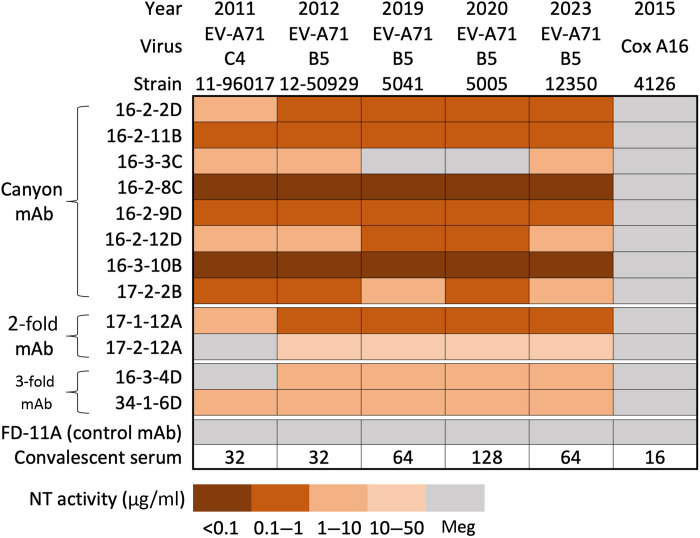
Neutralizing activities of anti–EV-A71 mAbs against recently circulating isolates (2019–2020 and 2023). Neutralizing (NT) activity of the antibodies was assessed using a cytopathic effect–based neutralization assay. Neutralizing concentrations are shown as a brown color gradient and expressed in microgram/milliliter. Genotype B5 EV-A71 virus that caused HFMD outbreaks in 2019–2020 (strains 5041 and 5005) and 2023 (strain 12350) in Taiwan were tested in the assay. Representative genotype C4 (strain 11-96017), genotype B5 (strain 12-50929), and coxsackievirus A16 (strain 4126) were also included. Convalescent serum that was collected from a patient with HFMD infected by EV-A71 in 2019 and anti–SARS-CoV-2 receptor-binding domain human IgG mAb FD-11A were used as controls. The neutralizing titer of the serum is expressed as the reciprocal of the dilution factor.

The majority of the 12 neutralizing antibodies have broad potency against EV-A71 strains of genotypes B4, B5, and C4 isolated between 1998 and 2016 (*14*). In the past decade, genotype B5 EV-A71 has caused major HFMD outbreaks in Thailand (2016–2017), Taiwan (2019), and Vietnam (2023) (*15*, *16*), reemerging in Taiwan and clustering phylogenetically with genotype B5 viruses circulating in other endemic regions (fig. S1). Eleven of the 12 mAbs retained comparable neutralizing activity against recent, 2019, 2020, and 2023 genotype B5 isolates (Fig. 1). The exception was 16-3-3C, which failed to neutralize the 2019 and 2020 B5 viruses but retained activity against the 2023 isolate. This loss of activity is likely due to the VP1 D164E substitution present in the 2019–2020 viruses but absent in the 2023 strain (fig. S2).

### Neutralizing antibodies bind in three clusters covering almost all the capsid surface

**Fig. 2.**
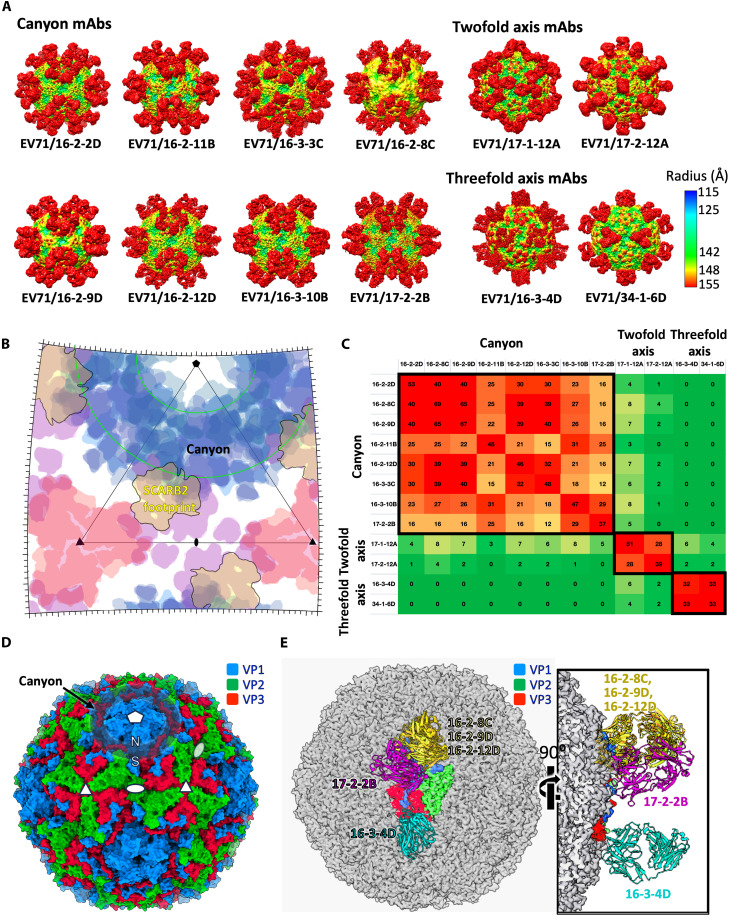
Cryo-EM reconstructions of EV-A71/Fab complexes and mapping of antibody footprints. (**A**) Cryo-EM reconstructions of EV-A71/Fab complexes. Genotypes B2 (strain MS742387), B5 (strain 12-96015), and C4 (strain FY/AH/CHN/2008) of EV-A71 were used for complex formation. The reconstructions are rainbow colored based on the distance (Å) of the surface from the center of the particle. (**B**) Schematic roadmap showing the footprints of anti–EV-A71 Fabs on the surface of the viral capsid in an asymmetric unit. Footprints of canyon binders are colored in blue, while two- and threefold axis binders are colored in purple and red, respectively. The footprint of the SCARB2 receptor is depicted in light brown. Footprints are drawn with some transparency, so that the colors are more intense in overlapping regions. The canyon region is outlined in green. (**C**) Heatmap showing the degree of epitope overlap. Numbers indicate the number of residues shared between Fab footprints. Green indicates no overlap, while red indicates major overlap. (**D**) Surface representation of the EV-A71 capsid with VP1, VP2, and VP3 colored in blue, green, and red, respectively. The positions of some fivefold, three, and twofold icosahedral symmetry axes are marked as pentagons, triangles, and ovals, respectively. The south (S) and north (N) walls of the canyon are also labeled. (**E**) Distinct binding patterns of IGHV4-39 antibodies on the EV-A71 capsid (gray) with a biological protomer highlighted (VP1 in blue, VP2 in green, and VP3 in red). Fabs 16-2-8C, 16-2-9D, and 16-2-12D are colored yellow; 17-2-2B in purple; and 16-3-4D in cyan.

To understand the functional activities and map the epitopes, Fabs were produced for all 12 mAbs and crystallized, and their structures were determined (table S2 and fig. S3). In addition, we solved cryo-EM structures of all 12 Fabs complexed with EV-A71 of subtype B2 (strain MS742387), B5 (strain 12-96015, very closely related to those that infected the three patients), or C4 (FY/AH/CHN/2008) (figs. S2 and S4A). The structures, resolved at 3.0 to 4.1 Å (fig. S4B), allowed mapping of the Fab-virus interactions in atomic detail despite the low occupancy of some Fabs (Fig. 2A, table S3, and representative density is shown in fig. S4C).

In all cases, the Fab bound to the native (N) antigenic state of the virus with footprints ranging from 929 to 1726 Å^2^ (table S4). Eight of the 12 antibodies (mAbs 16-2-2D, 16-2-8C 16-2-9D, 16-2-11B, 16-2-12D, 16-3-3C, 16-3-10B, and 17-2-2B) bound the canyon region encircling the icosahedral fivefold axes, while the remaining antibodies bind epitopes close to either the twofold (mAbs 17-1-12A and 17-2-12A) or threefold (mAbs 16-3-4D and 34-1-6D) axes (Fig. 2A). It was observed that 17-2-2B engages only a single protomer, while all other antibodies span two protomers (fig. S5).

Within each of these three binding groups, antibody footprints overlap, in line with the measured competition for binding (*14*), while antibodies targeting different regions share few or no common binding residues and hardly compete (Fig. 2, B and C). In total, the observed antibody footprints cover ∼85% of the residues exposed on the virus surface, leaving the area at the fivefold axes the only significant region not used (Fig. 2, A and B).

Canyon-binding antibodies interact mainly with VP1 (Fig. 2, B and D), although two of the eight are situated south of the canyon (defined in Fig. 2D) and contact all three surface proteins. In comparison, the twofold axis binders largely engage VP2, and threefold axis binders interact with both VP2 and VP3 (Fig. 2, B and D).

The heavy chains generally play the major role in Fab-EV-A71 interactions and tend to have a larger footprint on the capsid than the light chains (table S4), except for the southerly canyon binders. In one of these (17-2-2B), the heavy and light chains contribute to nearly equal areas of interaction, while in the other (16-3-10B), the light chain dominates (∼900 Å^2^ versus 500 Å^2^ for the light and heavy chain, respectively; table S4).

Conformational changes upon Fab binding were observed in most of the 12 EV-A71-Fab complexes. Eight showed changes in the complementarity-determining regions of the antibodies, and five displayed substantial changes in the EV-A71 capsid, mainly involving the VP1 B-C and G-H loops (table S5 and fig. S6). For four complexes (16-2-2D, 17-1-12A, 17-2-2B, and 17-2-12A), Fab binding induces conformational changes in the viral capsid to attain a better fit. In the fifth complex (16-2-11B), a major conformational switch in the VP1 B-C loop increases virus-antibody complementarity, thereby enhancing the binding. Overall, the movements in the viral epitope residues and the antibody paratope residues were broadly comparable with paratope shifts and usually less than 1 Å (table S5 and fig. S7A).

### A predominant gene family does not dictate a common binding pose

While a wide range of V, D, and J genes are used across the 12 antibodies, one heavy-chain gene family, IGHV4-39, is used by 5 of the 12 mAbs (16-2-8C, 16-2-9D, 16-2-12D, 17-2-2B, and 16-3-4D) (table S6). The preponderance of a single IGHV gene family occurs in other virus infections, such as the public IGHV3-53/66 responses against severe acute respiratory syndrome coronavirus 2 (SARS-CoV-2) (*17*–*18*), IGHV1-69 against influenza (*19*), and IGHV1-2/46 against HIV (*20*); for all of which, there is a single dominant antibody pose. In contrast, the IGHV4-39 gene family displays multiple modes of engagement with EV-A71, with binding sites widely distributed across the icosahedral asymmetric unit (Fig. 2E). Three antibodies (16-2-8C, 16-2-9D, and 16-2-12D) are from a single donor; have highly similar CDR-H3s; have a common IGHV4-39*01/IGHD22*01/IGHJ4*02 heavy-chain VDJ recombination (indeed 16-2-8C and 16-2-9D have identical CDR-H3s and likely share a clonal lineage); and, as expected, bind similarly within the canyon, although the light chain V gene usage differs for 16-2-12D (table S6). However, the remaining two show distinct binding modes; 17-2-2B is an aberrant southerly canyon binder with a short CDR-H3, and 16-3-4D binds at the threefold axis. We note also that a further IGHV4-39 binding antibody has been reported for EV-A71 (from a different donor), also binding at the threefold axis but in a quite different pose to 16-3-4D (*13*). For all of these alternative binding modes, the heavy-chain VDJ genes differ from those seen for 16-2-8C, 16-2-9D and 16-2-12D, so that we do not have evidence that the IGHV4-39*01/IGHD22*01/IGHJ4*02 recombination comprises a public clonotype.

### A long CDR3 enables neutralizing antibodies to access the canyon epitope

**Fig. 3.**
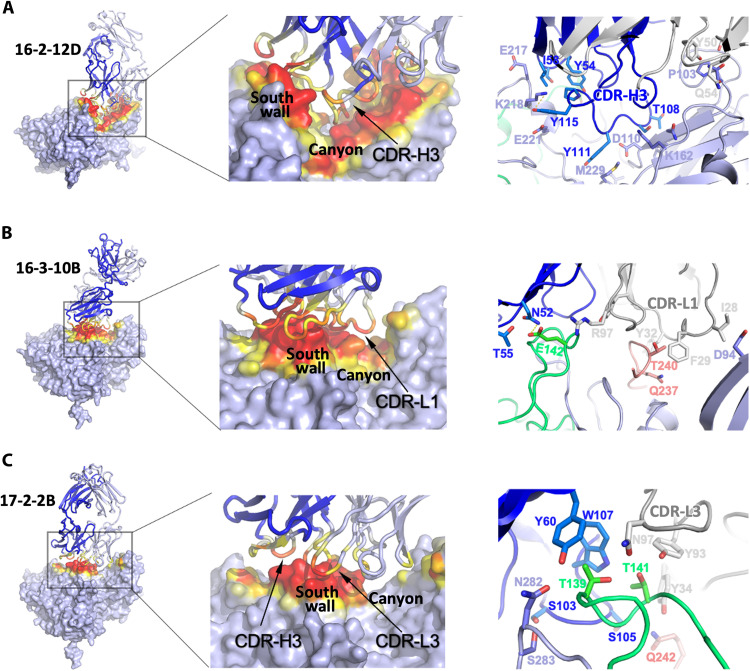
Varieties of canyon access by neutralizing antibodies. Different binding patterns of canyon-binding antibodies with EV-A71 are shown. (**A**) Fab 16-2-12D, representative of most canyon binders, inserts its CDR-H3 loop deeply into the bottom of the canyon region. (**B**) Fab 16-3-10B uses its extended CDR-L1 loop rather than CDR-H3 to reach into the canyon. (**C**) Fab 17-2-2B does not penetrate the canyon but engages along the southern wall of the canyon. Complexes were formed with EV-A71 B2 (strain MS742387) for 16-2-12D, C4 (strain FY/AH/CHN/2008) for 16-3-10B and B5 (strain 12-96015) for 17-2-2B. In the left panels, the EV-A71 protomer is shown as a gray surface, and the Fab is shown as a cartoon, with heavy and light chains colored in blue and gray, respectively. Contact areas between virus and Fab are highlighted in red for distances ≤4.0 Å and in yellow for >4.0 and ≤9.0 Å. In the right panels, VP1, VP2, and VP3 of EV-A71 are colored in light blue, green, and red, respectively. Heavy and light chains of the Fab are colored in dark blue and gray, respectively. Residues that involve the Fab-virus interactions and their neighboring residues are further indicated.

Canyon depressions encircle the fivefold axes of the otherwise relatively smooth EV-A71 capsid surface. Six of the eight canyon-binding antibodies have long CDR-H3 loops (four antibodies with ≥23 amino acids and two each with 18 and 15 amino acids) (Fig. 3A and table S7), enabling them to penetrate the canyon and interact with residues originally proposed to be concealed from antibody access (*21*). Intraloop disulfide bonds, found in four of the six longest CDR-H3 loops (table S7 and fig. S7B), presumably stabilize the loop and reduce the entropic penalty of binding. The 24-residue CDR-H3 loops have disulfide bridges in homologous positions, with four residues separating the two cysteines. Two antibodies, 16-2-8C and 16-2-9D, share identical sequences, and the third, 16-2-12D, has a very similar overall conformation despite having eight–amino acid differences. The distinctive conformation of the 15-residue loop of 16-2-11B is stabilized by a disulfide bond with three residues between the two cysteines (fig. S7B).

The southerly canyon binders are exceptions; they have the shortest CDR-H3s of all the mAbs (9 and 13 amino acids) and adopt entirely different binding poses compared to other canyon binders. In one of these antibodies (16-3-10B), its extended CDR-L1 reaches deep into the canyon, substituting for the role of the CDR-H3 (Fig. 3B). In contrast, the second antibody (17-2-2B) fails to penetrate the canyon; instead, its primary interactions, mediated by CDR-H3 and CDR-L3, occur along the southern wall of the canyon (Fig. 3C).

### Distinct binding modes of neutralizing antibodies allow for bivalent attachment

The twofold and threefold axis binders have somewhat shorter CDR-H3 loops than those of the major canyon-binding antibodies, with lengths of up to 16 amino acids (table S7). The two twofold axis binders exhibit distinct binding modes: 17-1-12A binds adjacent to the twofold axis, whereas 17-2-12A binds directly on the axis. The arms of immunoglobulin G (IgG) 17-1-12A may be long and flexible enough to span adjacent twofold axes and bind in a bivalent pattern (*22*). This cross-linking could stabilize the capsid in the native conformation, thereby preventing the capsid expansion required for genome release. The threefold axis binders also have distinct poses. Fab 34-1-6D binds on the axis, while 16-3-4D binds nearby. The distance between adjacent symmetry axes is compatible with the potential bivalent attachment of IgG molecules. Because of their binding at the symmetry axes, Fab 17-2-12A and Fab 34-1-6D can bind at most 30 and 20 molecules per capsid, respectively, whereas all other Fabs can occupy up to 60 sites per capsid—consistent with the relative Fab densities observed in the cryo-EM reconstructions.

### Nearly all neutralizing antibodies block SCARB2

**Fig. 4.**
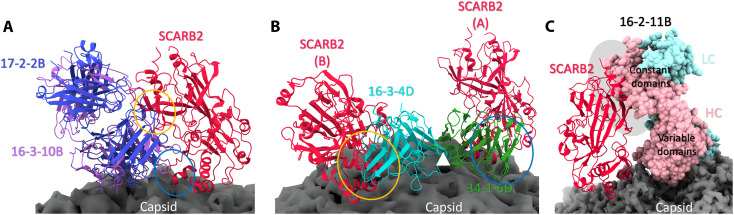
Clashes between the SCARB2 receptor and neutralizing antibodies. (**A**) Canyon-binding antibodies 17-2-2B and 16-3-10B, representative of the majority of canyon binders, overlap with the SCARB2 receptor. Yellow and blue ovals highlight the regions of antibody-receptor clash. The EV-A71 virion is shown as a gray surface, and the Fab is shown as a cartoon, with 17-2-2B, 16-3-10B, and SCARB2 colored in blue, purple, and red, respectively. (**B**) Threefold axis-binding antibodies sterically block SCARB2 engagement. SCARB2 (A) and (B) denote two receptor molecules bound to neighboring protomers. The white triangle shows the approximate position of the threefold icosahedral symmetry axis. Yellow and blue ovals highlight the regions of antibody-receptor clash. EV-A71 is shown as a gray surface and the Fab is shown as a cartoon, with 16-3-4D, 34-1-6D, and SCARB2 colored in cyan, green, and red, respectively. (**C**) Constant domain of the heavy chain of antibody 16-2-11B clashes with SCARB2, as indicated by the gray oval. EV-A71 is shown as a gray surface. 16-11B heavy chain, light chain, and SCARB2 are colored in pink, cyan, and red, respectively.

Blocking cellular receptor attachment is a common mechanism of virus neutralization; in line with this, we noted that the mAbs neutralize EV-A71 more efficiently when administered at the preattachment stage (*14*). We therefore examined potential overlap between the footprints of the Fabs with that of the internalization receptor SCARB2 (Fig. 2B and table S8). Seven of the eight canyon-binding antibodies overlap SCARB2 (Fig. 4A), the exception being 16-2-11B, as do both twofold axis binders, indicating that approximately two-thirds of the antibodies directly compete with SCARB2.

In contrast, the threefold axis binders share no common binding residues with SCARB2 (Fig. 2B); however, when the Fab-virus complexes are superimposed onto the SCARB2-virus complex (Fig. 4B), both Fabs sterically clash with the receptor. Specifically, 34-1-6D impinges on SCARB2 bound to the same protomeric unit, whereas 16-3-4D clashes with a receptor bound to a neighboring protomer. Nevertheless, because 34-1-6D can occupy no more than 20 sites on the capsid, it can block at most one in three potential receptor binding sites.

Last, although the variable domains of Fab 16-2-11B do not overlap the SCARB2 footprint, the constant domain of the heavy chain does (Fig. 4C), potentially abrogating SCARB2 attachment. However, if the antibody has sufficient flexibility to allow simultaneous engagement of both arms, then an alternative mechanism of neutralization may be at work.

### Most antibodies slightly increase virion stability, while some induce empty particles

Antibody-induced premature genome release represents one mechanism of enterovirus neutralization (*11*). To assess the effect of Fab binding on virion stability, we measured the melting temperature of EV-A71 B5 particles (strain 12-96015) at increasing Fab/virus molar ratios. For most Fabs, increasing the ratio from 6 to 600 resulted in a modest rise in melting temperature by about 0.5° to 1°C, suggesting that Fab binding slightly stabilizes the particle (fig. S8). This is in line with our structural data, since all Fabs except 17-2-2B interact with more than one protomer of the capsid (fig. S5), potentially stabilizing the capsid.

**Fig. 5.**
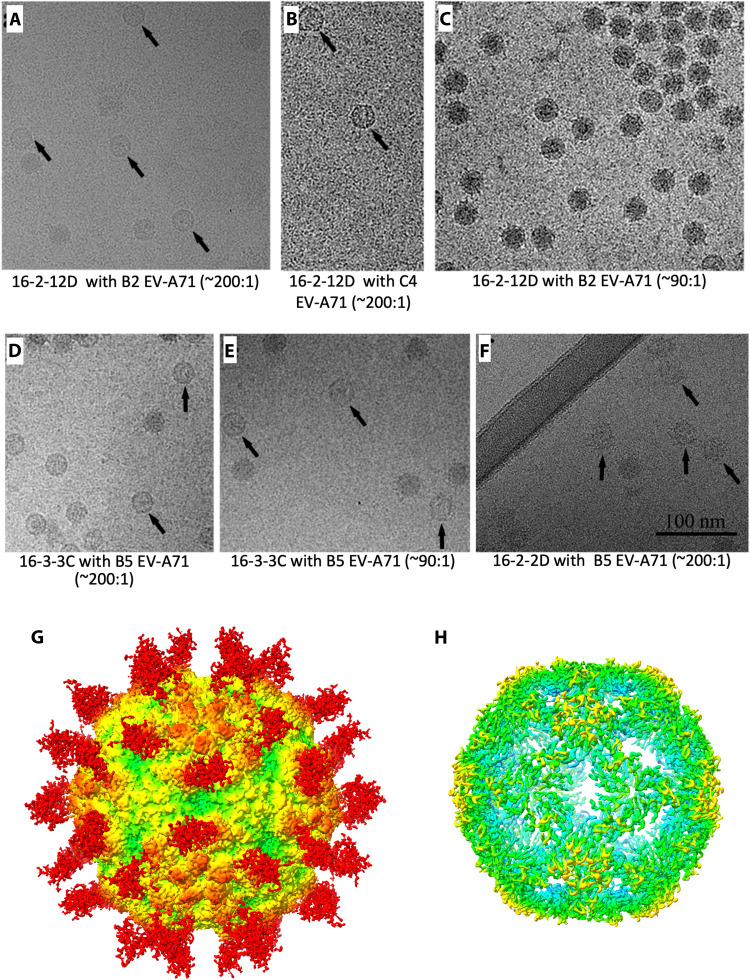
Empty particles of EV-A71 induced by Fabs 16-2-12D, 16-3-3C, and 16-2-2D. (**A** and **C**) EV-A71 genotype B2 (MS742387) mixed with Fab 16-2-12D. (**B**) EV-A71 genotype C4 (FY/AH/CHN/2008) mixed with Fab 16-2-12D. (**D** and **E**) EV-A71 genotype B5 (12-96015) mixed with Fab 16-3-3C. (**F**) EV-A71 genotype B5 (12-96015) mixed with Fab 16-2-2D. Molar ratio of Fab: EV-A71 particle in (A), (B), (D), and (F) is ∼200:1 and in (C) and (E) is ∼90:1. Empty particles can be observed in all of these micrographs except (C). (**G**) Cryo-EM reconstruction of EV-A71 empty particle complexed with Fab 16-3-3C. The map is shown at low contour level so the weak density of Fab 16-3-3C can be seen. Density for the EV-A71 empty particle in this complex is shown in (**H**) at high contour level, revealing the gaps in the capsid characteristic of the expanded particle. The maps are rainbow colored in the same way as in Fig. 2A.

Fab 16-2-2D is an exception, decreasing the melting temperature by ∼2.5°C at high Fab concentrations. We next incubated virus with excess of Fab (3.3 Fabs per binding site) and found that Fab 16-2-2D induced the formation of EV-A71 empty particles after 5-min incubation at 37°C (Fig. 5). Extending the analysis to all antibodies revealed that Fabs 16-2-12D and 16-3-3C also triggered genome release, producing significant numbers of empty particles even after less than 10-s incubation on ice, although sufficient intact particles remained to allow structure determination (Fig. 5). Notably, Fab 16-3-3C was able to produce empty particles at only ∼1.5 Fabs per binding site (Fig. 5).

Thus Fabs 16-2-2D, 16-2-12D and 16-3-3C can induce genome release, inactivating the virus and converting the capsid to the expanded H antigenic conformation (Fig. 5) (*5*). For instance, for 16-3-3C, 96% of the particles passing two-dimensional (2D) classification were empty (whereas the number of empty particles with nonuncoating mAbs was negligible). The structure determination of this 16-3-3C/EV-A71 complex confirmed that the empty particle was in the H antigenic conformation (table S3) and demonstrated that 16-3-3C recognizes nearly the same epitope on both the H and N antigenic forms.

However, the Fab density observed in the H antigen complex was much lower than that in N antigen complex, consistent with a lower binding affinity for the expanded EV-A71 particle (unfortunately, the map for this complex is at too low a resolution for us to offer a structural explanation for the loss of affinity). For Fab 16-2-2D, the particle conversion is consistent with the destabilization observed upon complex formation, while the reason why the other two uncoating Fabs, 16-2-12D and 16-3-3C, did not reduce virus stability remains unclear.

### The antibodies have higher affinity for full particles of EV-A71

Binding kinetics measured by biolayer interferometry (fig. S9, A and B, and table S8) showed that the Fabs exhibit differing dissociation constants (*K*_D_s) between the N and H antigenic forms of EV-A71 B5 (strain 12-96015). It is notable that all Fabs except 16-3-4D bind the native virion more tightly, often with affinities about one log higher (fig. S9). This likely reflects that these antibodies, derived from infected patients, were elicited against intact, infectious EV-A71 particles. The difference in affinity between full and empty particles was not uniform across Fabs, suggesting that structural rearrangements during capsid expansion affect individual epitopes to varying extents (fig. S9).

### Escape mutants evade neutralization both directly and indirectly

**Fig. 6.**
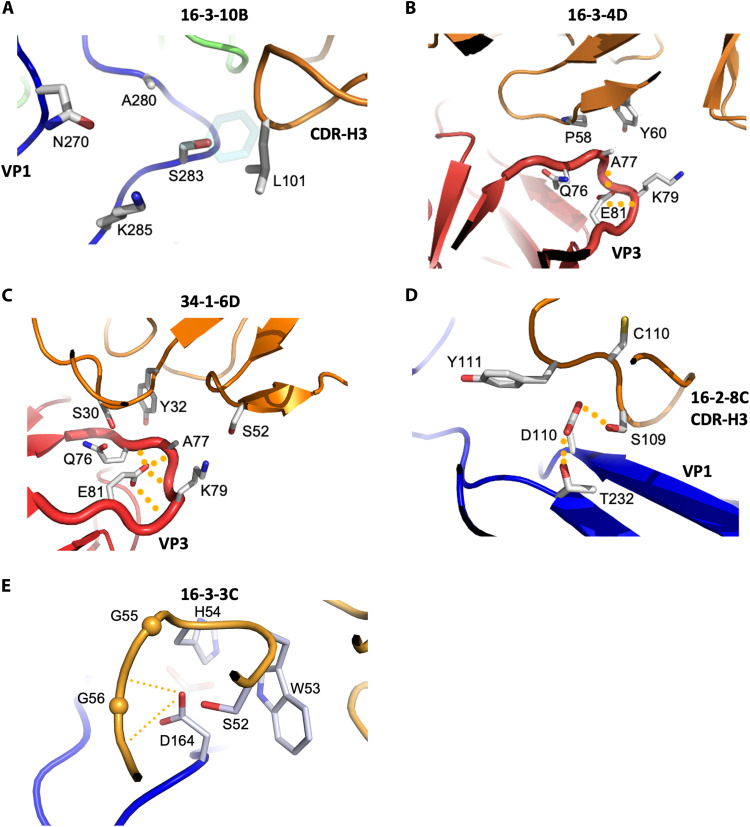
Molecular mechanism of escape mutations. (**A**) Substitution VP1 S283F confers escape of genotype C4 EV-A71 from antibody 16-3-10B. The large phenylalanine side chain (shown as cyan semitransparent sticks) introduced reduces the complementarity with antibody 16-3-10B. (**B** and **C**) Substitution VP3 E81G/K confers escape of genotype B5 EV-A71 from antibodies 16-3-4D (B) and 34-1-6D (C). Although residue VP3 E81 is not directly involved in antibody binding, it stabilizes a nearby loop (shown thicker) that is targeted by antibodies 16-3-4D and 34-1-6D. The observed escape mutations VP3 E81G/K destabilize this loop, interfering with antibody binding. (**D**) Substitution VP1 T232A confers escape of EV-A71 from antibodies 16-2-8C, 16-2-9D and 16-2-12D. In the wild type, VP1 T232 interacts with VP1 D110, making the side chain of VP1 D110 optimally interacts with antibody 16-2-8C, 16-2-9D, or 16-2-12D. The mutation VP1 T232A disrupts the interaction between VP1 232 and VP1 D110, increasing flexibility of the side chain of VP1 D110 and leading to escape from antibodies. (**E**) Substitution VP1 D164E confers escape of genotype B5 EV-A71 from antibody 16-3-3C. The longer side chain of glutamic acid (shown as semitransparent sticks) introduced by this substitution reduces the complementarity with antibody 16-3-3C. Hydrogen bonds are indicated by orange dashed lines. In the figure, VP1, VP2, and VP3 of EV-A71 are colored in blue, green, and red, respectively, while Fabs are colored in orange.

Escape mutants were selected in vitro to identify the residues critical for binding and neutralization by the 12 anti–EV-A71 mAbs (table S9) (*14*). The mutations are spread across the capsid surface (fig. S10). Only one substitution, at VP2 residue 149, is within the SCARB2 binding site. This residue is not conserved across sub genotypes and therefore unlikely to be essential for receptor binding (fig. S2). In contrast, several mutations are grouped around the edge of the receptor footprint. As shown in fig. S10, most mutations lie within the footprint of one or more antibodies, clustering densely within the canyon region where most antibodies bind. These mutations likely impair antibody binding directly. Approximately half of mutations involve either a loss or gain of charge, while most of the remainder involve a substantial change in the bulk of the side chain (*23*). One example is the VP1 S283F mutation, located within the binding site of antibody 16-3-10B. The large phenylalanine side chain occupies the space normally filled by CDR-H3 L101 of the Mab, thereby sterically hindering attachment of the antibody (Fig. 6A). Nonetheless, comparing the observed substitutions with the expected distribution of random residue changes using the BLOSUM62 matrix (*23*) showed no evidence that the mutations were selected to maximize physicochemical differences.

For 5 of the 12 antibodies, escape-conferring substitutions lie outside the antibody footprint and probably act indirectly by altering the presentation of binding residues. For example, the EV-A71 VP3 E81G/K mutation abrogates binding of antibodies 16-3-4D and 34-1-6D. Although this residue does not directly interact with either antibody, the side-chain carboxylate stabilizes a preceding loop engaged by both. Substitution to either glycine or lysine likely destabilizes the loop, thereby interfering with antibody binding (Fig. 6, B and C). Another example is VP1 T232A mutation, where the threonine side chain interacts with VP1 D110 to maintain a conformation that optimizes key interactions with mAbs 16-2-8C, 16-2-9D and 16-2-12D. Substitution to alanine impairs the interaction with VP1 D110, increasing local flexibility, and subsequently leads to viral escape (Fig. 6D). In the study, we formed complexes of 34-1-6D, 16-2-9D or 16-2-12D with EV-A71 strain MS742387 (genotype B2) because it does not have the above substitutions and is neutralized by these antibodies.

Nine of the 12 mAbs exhibited broad potency against clinical strains of genotypes B4, B5, and C4 (*14*) despite the observation that all surface mutations between these genotypes lie within the footprint of at least one mAb (fig. S2). Antibody 16-3-3C cannot neutralize certain clinical strains of B4 and a subset of B5 strains (Fig. 1). We postulate that this is due to the VP1 D164E substitution in these strains. The aspartic acid side chain interacts with mAb 16-3-3C by hydrogen bonding with the side chain of S52 and the amino group of G56 from the CDR-H2, interactions that would be disrupted by the longer glutamate side chain (Fig. 6E). The critical role of this interaction to neutralization by 16-3-3C is underscored by the loss of this aspartate in several escape mutations (D164G/N/V) selected in vitro (table S9).

None of the antibodies neutralize EV-A71 C2 strains 98-2086 (GenBank AAD22046.3) and 99-1691 (GenBank ADR70728.1) (*13*). Although most antibodies also fail to neutralize the C1 strain 98-4215 (GenBank AFN20476.1), a subset neutralize both this earlier and more recently circulating C1 strains with variable potencies (table S1) (*24*). The capsid surface is rather conserved among subtypes, with only 10– of 28–amino acid substitutions mapping to the surface of the capsid, and these tend to involve residues with similar side chains (fig. S2). Since none of the 10 surface substitutions lie within the epitopes of all 12 mAbs, resistance observed in these C1 and C2 strains cannot be attributed to a single mutation.

### Antibodies provide both prophylactic and therapeutic protection In vivo

**Fig. 7.**
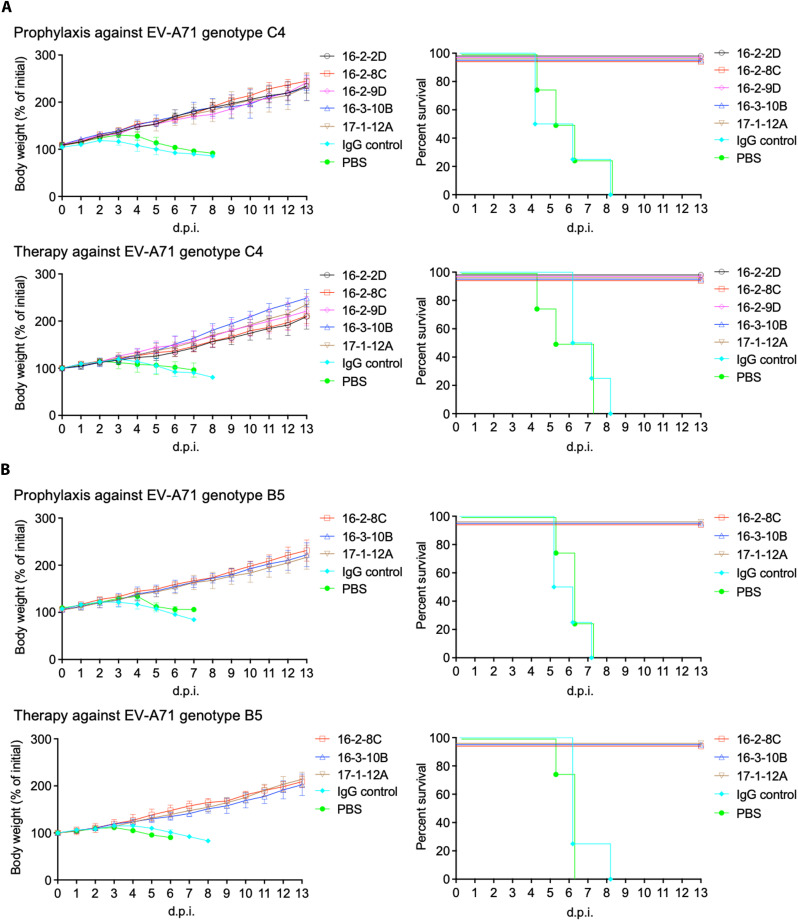
In vivo protection by neutralizing anti–EV-A71 mAbs in the human SCARB2 mouse model. (**A**) Prophylactic and therapeutic efficacy of antibodies (10 mg/kg) against genotype C4 EV-A71. A single dose of antibody or control was administered intraperitoneally 24 hours before or after a lethal challenge of genotype C4 EV-A71 12-73 (*n* = 4 per group). (**B**) Prophylactic and therapeutic efficacy of antibodies (10 mg/kg) against genotype B5 EV-A71. A single dose of antibody or control was administered intraperitoneally 24 hours before or after a lethal challenge of genotype B5 EV-A71 13-50144 (*n* = 4 per group). Anti-influenza B hemagglutinin IgG mAb (BN-2B) was used as a human IgG control. Weight changes and survival rate were monitored following infection. The weight data represents the means ± SD days post infection (d.p.i.).

Given their effectiveness in vitro and the distinctive epitopes they target, we examined prophylactic and therapeutic efficacy in the human SCARB2-transgenic mice model (*25*). In a control experiment, 3-week-old human SCARB2-transgenic mice were infected intraperitoneally with a lethal (1.5 × 10^5^ plaque-forming units) dose of genotype C4 12-73 or genotype B5 13-50144 EV-A71. The mice developed weight loss and/or motor deficit at days 3 to 7 postinfection and died within 2 weeks (Fig. 7) (*13*).

In a prophylactic experiment, a single dose of canyon-binder mAbs 16-2-2D, 16-2-8C, 16-2-9D, 16-3-10B, twofold binder mAb 17-1-12A, or an isotype control antibody was administered intraperitoneally (10 mg/kg) 24 hours before challenge. While mice treated with isotype control or phosphate-buffered saline (PBS) developed weight loss and 100% mortality after challenge, all neutralizing antibodies conferred complete protection from weight loss and mortality after challenge (Fig. 7, A and B). In a therapeutic experiment, a single dose of neutralizing antibody or isotype control was administered intraperitoneally (10 mg/kg) 24 hours after challenge. Again, in all cases, the neutralizing antibodies completely prevented both weight loss and mortality upon infection (Fig. 7, A and B), whereas mice challenged after treatment with isotype control or PBS developed weight loss and death.

## DISCUSSION

We present data on a diverse set of potent and broadly neutralizing anti–EV-A71 mAbs derived from three pediatric patients that exhibit antigenic footprints covering almost the entire viral surface. Eleven of the 12 antibody footprints span two protomers, a similar concentration of antigenic sites at interfaces has been reported for poliovirus (*26*), suggesting that this may be a common feature for enterovirus antigenic sites. Despite the epitopes being broadly distributed on three distinct regions—the canyon, twofold axis, and threefold axis—all antibodies clash with the viral receptor SCARB2 when interacting with the EV-A71 capsid, indicating their potential to block receptor engagement. If the kinetics of binding allows them to outcompete SCARB2, then this would lead to virus neutralization. We also find that three Fabs—16-2-2D, 16-2-12D and 16-3-3C—induce genome release, representing another potential mechanism of neutralization. All three antibodies that induce genome release are canyon binders, and they may trigger conformational switching to the H form in an analogous manner to the uncoating receptors of EV-A71 (*8*). However, it has been reported that a mouse-derived antibody, E18, can convert infectious virions of EV-A71 into empty particles by binding near the threefold icosahedral axes (*11*), demonstrating that there are multiple triggers for uncoating. Last, none of the 12 Fabs markedly increased the melting temperature of the EV-A71 virion, suggesting that particle stabilization is not a major neutralization mechanism.

All 12 mAbs bind strongly to EV-A71 native (N) antigenic form particles, and most have weaker binding to expanded (H) particles. This is expected, since by analogy with poliovirus, H particles would be expected to have different immunogenicity (*27*); indeed, poliovirus expanded particles fail to elicit a protective immune response (*28*). In line with this, EV-A71 virus-like particles in the N antigenic conformation (*5*, *29*) elicit potent immune responses to protect mouse against lethal challenge, whereas H particles elicit much less protection (*30*). In apparent contrast to this, Kingston *et al.* (*31*) found that both thermally stabilized N-form and H-form virus-like particles elicited neutralizing antibodies, although it was unclear whether the native conformation provides greater protection in vivo.

We note that expanded particles are produced alongside native particles when live virus is purified from mammalian cells, potentially reducing the immunologically relevant portion of purified vaccine antigen. By analogy with poliovirus (*32*), thermally stabilized EV-A71 particles might therefore be a promising option for future vaccines (*27*).

The structural convergence of neutralizing antibodies on common epitopes and the use of public gene families have been observed for viruses including SARS-CoV-2 (*17*, *18*), influenza (*19*), and Junín virus (*33*). In the case of EV-A71, we also find that significant epitope convergence arises from the use of the same germline gene, but that it can also arise from totally different genes. Structural convergence has been taken to suggest that the mAbs are selected by V(D)J recombination and somatic hypermutation for the same purpose, i.e., to potently neutralize the virus with a common mechanism. The structurally convergent mAbs in this paper—similar to those against SARS-CoV-2, influenza, and Junín virus—do indeed block virus-receptor interactions. In addition, we also observe strong apparent germline convergence, with the same heavy-chain variable gene family IGHV4-39, used by 5 of the 12 antibodies, and by two of the three individuals. However, we find that this does not dictate epitope convergence because while three of these antibodies (16-2-8C, 16-2-9D and 16-2-12D) bind in a highly similar pose at the canyon, another, 17-2-2B, has a much shorter CDR-H3 loop and binds a quite different site within the canyon, and the final mAb (16-3-4D) binds a completely different epitope at the threefold axis. This demonstrates that antibody-antigen interactions are complex and not fully dictated by the heavy-chain variable gene family.

Several mouse-derived anti–EV-A71 antibodies have similar binding patterns to the human antibodies studied here. For example, antibody 22A12 binds at the canyon region (PDB: 3 J91) (*34*), D5 at the twofold axis area (PDB: 3JAU) (*9*) and R10 at the threefold axis area (PDB: 5ZUF) (*35*). In addition, antibody MA28-7, targeted a distinct site at the fivefold vertex, not observed for human antibodies (PDB: 3J3Z) (*36*). Although the immunization of animals including mouse has been widely used to understand the immune response to viral infection, the antibody responses are significantly different. Most notably, mouse antibodies have shorter CDR-H3 loops, which would be unlikely to penetrate the EV-A71 canyon in the way that is commonly used by the human antibodies studied here. This is in line with a recent study on coxsackievirus B3 reported that mouse antibody responses were more restrictive than those in humans (*37*). We suggest that mouse models are of limited value for understanding human B-cell responses against such viruses. Although there has been considerable progress in the use of AI methods for predicting protein structure (*38*, *39*), antibodies remain especially challenging, and the substantial set of structural data presented here may contribute to improving the algorithms.

Last, the antibodies described here effectively protect against lethal challenge in a susceptible mouse model, underlining the potential therapeutic value of potent and broadly reactive human mAbs in clinical applications. Furthermore, a cocktail of antibodies that target distinct, nonoverlapping epitopes demonstrated enhanced potency in the neutralization matrix assay against EV-A71 strain 12-96015 (fig. S11). This increased potency indicates synergy between the canyon-binding 16-2-9D and either the twofold axis-binding 17-1-12A or the threefold axis-binding 34-1-6D. These findings imply that targeting discrete binding sites may facilitate the formulation of a cocktail of antibodies and warrants further investigation into in vivo synergy and the prevention of viral escape in future studies.

## MATERIALS AND METHODS

### Fab preparation from IgG

Fabs 16-2-9D and 17-2-2B were generated from whole IgGs by papain digestion following the Pierce Fab Preparation Kit protocol (Thermo Fisher Scientific).

### Fab cloning expression and purification

Full details are presented in (*40*). In brief, plasmids containing V-D-J region DNA and V-J region DNA were ordered from GeneArt, amplified by polymerase chain reaction (PCR), and cloned directly into the expression plasmids using ligation independent cloning (In-Fusion Cloning, Bimake). Three expression plasmids were constructed containing resident human antibody heavy- or light-chain constant regions to produce recombinant Fab fragments with inserted human antibody VH or VL sequences as previously described for mouse Fabs (*41*). The vector pOPINhuVH was used for heavy-chain genes, pOPINhuVLkappa for kappa light-chain genes, and pOPINhuVLlambda for lambda light-chain genes [these have been deposited with Addgene, IDs 234057 (pOPINHuVH), 234058 (pOPINHuVLkappa), and 234059 (pOPINHuVLlambda)]. The pOPINhuVH vector encodes a C-terminal 6*His tag for subsequent protein purification. Recombinant plasmids were verified by Sanger sequencing.

Human embryonic kidney (HEK) 293T cells were cultured in roller bottles (Greiner Bio-One) at 37°C in 200 ml of Dulbecco’s modified Eagle’s medium (DMEM) supplemented with 10% fetal bovine serum (FBS), 1% 100× l-glutamine, and 1% 100× minimum essential medium (MEM) + nonessential amino acids (NEAA). When the cells were confluent, the medium was removed, and new medium (200 ml of DMEM supplemented with 2% FBS, 1% 100× l-glutamine, and 1% 100× MEM + NEAA) was added. At the same time, 0.25 mg of heavy-chain plasmid, 0.25 mg of light-chain plasmid, 50 ml of DMEM, and 1 ml of polyethyleneimine (1 mg/ml) were mixed, incubated at room temperature for 10 min, and transferred to each roller bottle. Cells were incubated at 37°C and harvested after 4 days. The medium was spun at 1000*g* for 10 min, the supernatant was filtered to remove cell debris and dialyzed in 15 liter of buffer [23.2 mM Na_2_HPO_4_, 1.7 mM NaH_2_PO_4_, and 250 mM NaCl (pH 8.0)]/1 liter of supernatant at 4°C. After 24 hours, the buffer was replaced, dialysis was continued for another day, and the supernatant was filtered. Nickel columns (5 ml Cytiva) were used for affinity purification. Filtered Fab samples were loaded onto the column, washed with ∼50 ml of buffer [20 mM tris (pH 8.0), 200 mM NaCl, and 30 mM imidazole], and Fab was eluted in 5 ml of elution buffer [20 mM tris (pH 8.0), 200 mM NaCl, and 500 mM imidazole]. Fabs were further purified by size exclusion chromatography on a Hiload 16/60 Superdex 75 column (Cytiva) using 20 mM tris, 200 mM NaCl, and pH 8 running buffer; concentrated using a 10-kDa centrifugal filter (Sigma-Aldrich); and stored at 4°C. Fab 16-3-3C required further purification using an HiTrap SP HP ion-exchange column (Cytiva).

### Crystallization

Fab crystals were grown in CrystalQuick X plates (Greiner Bio-One) at 20°C via sitting-drop vapor diffusion, with 100 nl of Fab plus 100 nl of precipitant dispensed with a Cartesian robot (*42*). Drops were imaged using a Formulatrix Rock Imager 1000. Microseeding (*43*) improved crystal quality for Fabs 17-1-12A and 16-2-12D. The crystallization concentrations and the conditions yielding good Fab crystals were: 16-2-2D (51.7 mg/ml): 30% (v/v) pentaerythritol ethoxylate (15/4 EO/OH), 0.05 M (NH_4_)_2_SO_4_, and 0.1 M bis-tris (pH 6.5); 16-2-8C (26.4 mg/ml): 25% (w/v) PEG-3350 (polyethylene glycol, molecular weight 3350), and 0.1 M bis-tris (pH 6.5); 16-2-9D (12.6 mg/ml): 25% (w/v) PEG-3350 and 0.1 M citrate (pH 3.5); 16-2-11B (45.9 mg/ml): 20% (w/v) PEG-3350 and 0.2 M NaCOOH; 16-2-12D (49.9 mg/ml): 20% (w/v) PEG-3350 and 0.2 M NaH_2_PO_4_; 16-3-3C (32.1 mg/ml): 25% (w/v) PEG-3350 and 0.1 M bis-tris (pH 5.5); 16-3-4D (50 mg/ml): 2% (v/v) PEG-400, 2 M (NH_4_)2SO_4_, and 0.1 M Hepes-Na (pH 7.5); 16-3-10B (35.3 mg/ml): 30% (w/v) PEG-6000 and 0.1 M Hepes (pH 7.0); 17-1-12A (19.8 mg/ml): 1.5 M Li_2_SO_4_ and 0.1 M Hepes-Na (pH 7.5); 17-2-2B (14.4 mg/ml): 30% (w/v) polyethylene glycol monomethyl ether 2000, 0.1 M CH_3_COONa (pH 4.6), and 0.2 M (NH_4_)_2_SO_4_; 17-2-12A (30.2 mg/ml): from 20% (w/v) PEG 3350, 0.2 M di-ammonium hydrogen citrate, or 25% (w/v) PEG-3350 and 0.1 M citrate (pH 3.5); and 34-1-6D (44.7 mg/ml): 30% (w/v) PEG 3000 and 0.1 M 2-(cyclohexylamino)ethanesulfonic acid (pH 9.6).

### Crystallographic data collection and structure determination

Crystals were immersed in reservoir solution supplemented with 25% (v/v) glycerol or ethylene glycol before flash-freezing in liquid nitrogen. X-ray diffraction data were recorded (at 100 K) as 0.1° rotations at I03, I04, or I24 beamlines at Diamond Light Source (Didcot, Oxford) using a Pilatus detector. Data were indexed and integrated with Xia2 dials or 3dii (*44*), and data for the same Fab with the same space group and similar unit cell dimensions merged where appropriate. MOLREP (*45*) or Phaser-MR (*46*) were used for molecular replacement, and REFMAC (*47*) and PHENIX (*48*) were used for structure refinement. Model building used COOT (*49*). Data collection and refinement statistics are shown in table S2. Figures were prepared using PyMOL (*50*) (Schrödinger, LLC).

### Virus production

Full details are presented in (*40*). In brief, Vero and rhabdomyosarcoma cells were cultured in T175 flasks using 20 ml of DMEM supplemented with 10% FBS, 1% 100× l-glutamine, and 1% 100× MEM + NEAA, respectively. At ∼70 to 80% confluence, the medium was removed, and 20 ml of new medium (DMEM with 2% FBS, 1% 100× l-glutamine, and 1% 100× MEM + NEAA) was added. Cells were infected with EV-A71 genotype B2 (strain MS742387), EV-A71 B5 (strain 12-96015) or EV-A71 C4 (FY/AH/CHN/2008, isolated from Fuyang, Anhui Province, China in 2008) at 37°C. Cells were left in the incubator for 24 hours after cell death to ensure complete release of the virus. Samples containing virus were harvested, mixed with PBS supplemented with 0.5% NP-40, and freeze-thawed three times before adding deoxyribonuclease to a final concentration of 50 U/ml. Cell debris was removed by centrifugation at 3600*g* for 30 min, and 8% (w/v) PEG-6000 was dissolved in the supernatant. Samples were stored overnight at 4°C and then centrifuged at 3600*g* for 1 hour. The pellet was dissolved in 30 ml of buffer [50 mM Hepes (pH 7.8) and 200 mM NaCl] and centrifuged at 3600*g* for 30 min. Virus in the supernatant was then spun through a sucrose cushion [50 mM Hepes (pH 7.8), 200 mM NaCl, and 30% (w/v) sucrose buffer] at 103,000*g* for 3 hours using a Beckman SW 32 Ti rotor. A 0.5 ml of buffer [50 mM Hepes (pH 7.8) and 200 mM NaCl] was added to the pellet, which was stored overnight at 4°C, then dissolved, and centrifuged twice at 16,000*g* for 30 min to remove undissolved debris. Final purification used a sucrose gradient [50 mM Hepes (pH 7.8), 200 mM NaCl, 15% (w/v) sucrose, 50 mM Hepes (pH 7.8), 200 mM NaCl, and 45% (w/v) sucrose] centrifuged at 103,000*g* for 3 hours with a Beckman SW 32 Ti rotor. Bands for full and empty particles were harvested separately, applied to a desalting column to remove sucrose, and concentrated using 100-kDa cutoff filters (Sigma-Aldrich).

Negative-stain electron microscopy was used to check virus particles before cryo-EM. Particles were added to glow-discharged, formvar/carbon-coated 300-mesh copper grids (Agar Scientific) and incubated for 1 min, and excess sample was blotted away with filter paper before washing three times with deionized water, staining with 1% uranyl acetate for 1 min, removing excess stain and examining on an FEI T12 electron microscope (Thermo Fisher Scientific).

### Cryo-EM sample preparation, data collection, and analysis

For full details, see (*40*). In brief, purified virus and Fab were mixed and incubated for 1 to 10 min on ice or at 37°C at a capsid/Fab molar ratio of 1:300. 4 μl of sample was loaded on glow-discharged holey carbon-coated copper grids (CF-2/1-2C, Protochips) or ultrathin carbon grids (AG01824, Agar Scientific), incubated at room temperature for at least 10 s, blotted for 3 to 4 s, and vitrified in liquid ethane (Cryoplunge 3, Gatan).

EM data were collected at 300 kV on a Tecnai F30 Polara microscope (Thermo Fisher Scientific) equipped with a Gatan GIF Quantum energy filter (30-eV slit width) Gatan K2 direct electron detector. Data were recorded as movies (24 to 40 frames, each 0.2 to 0.25 s) using SerialEM (*51*), in super-resolution mode with a defocus range from −0.5 to −2.5 μm. The calibrated magnification was ×37,037, corresponding to a pixel size of 1.35 Å. The dose rate was 4 to 6 e^−^/Å^2^ per s, resulting in a total electron dose of 22 to 30 e^−^/Å^2^. Data collection statistics are shown in table S3.

Movie frames were aligned using MotionCor2 (*52*) and the contrast transfer function corrected with CTFFIND3 (*53*). Micrographs with astigmatism or significant drift were discarded. Particles were picked automatically using ETHAN (*54*) and manually screened in EMAN2 (*55*). Reconstruction used the gold-standard refinement procedure in Relion 2 and 3.1 (fig. S4A and table S3) (*56*). Reference-free 2D class averaging was followed by reference-based 3D classification and refinement using an initial model of EV-A71 [PDB: 3VBH (*5*)], filtered to 50-Å resolution.

The crystal structure of EV-A71 [PDB: 3VBH (*5*)] and the crystal structures of Fabs were fitted into the cryo-EM maps (with the amino acid sequence of EV-A71 updated as required) using COOT. In some cases, the density for the Fab was weak but did not preclude modeling. Models were further refined using Phenix.real_space_refine (*57*) (table S3). EV-A71-Fab interface residues were identified with PISA (*58*). Roadmap representations were calculated using Rivem (*59*). Figures were prepared using UCSF Chimera (*60*) and PyMOL (*50*) (Schrödinger LLC).

### Assay of thermal stability and Fab affinity

For full details, see (*40*). In brief, the PaSTRY thermal stability assay (*61*) used semi skirted 96-well PCR plates (4Titude). Each well contained 0.3 μg of EV-A71 B5 (strain 12-96015) and SYTO9 stain (Thermo Fisher Scientific) to a final concentration of 5 μM. The effect of Fab on particle stability was measured using 0:1 (control), 6:1, 20:1, 60:1, 200:1, and 600:1 molar ratios of Fab:EV-A71 particles in 20 mM tris and 200 mM NaCl (pH 7.4) buffer, buffer being added to each well to 50-μl final volume. Plates were sealed, spun for 2 min at 500*g*, and heated from 25° to 99°C, at 1°C/min, in an Mx3005p quantitative PCR machine (Stratagene, Agilent Technologies). Fluorescence changes were monitored with excitation and emission wavelengths of 492 and 585 nm, respectively, and data were analyzed using JTSA (http://paulsbond.co.uk). Experiments were performed in triplicate.

Binding kinetics were assayed at 30°C on an Octet RED96e (Sartorius) to obtain *K*_D_s of Fabs for full and empty particles of EV-A71 B5 (strain 12-96015). EV-A71 particles were attached to AR2G biosensors using the manufacturer’s protocol (Sartorius). Biosensors were equilibrated in deionized water for at least 10 min before activating in 20 mM 1-ethyl-3-[3-dimethylaminopropyl] carbodiimide hydrochloride and 10 mM *N*-hydroxysulfosuccinimide for 300 s. Purified EV-A71 full or empty particles in 10 mM sodium acetate buffer (pH 6.0) were then immobilized to the sensors by soaking for 10 min (final particle concentration of ∼200 μg/ml). Biosensors were then quenched in 1 M ethanolamine (pH 8.5) for 300 s and washed with 20 mM tris and 200 mM NaCl (pH 7.4) buffer for 1 min. Serial dilutions (2×), bracketing the expected dissociation constant of each Fab, were associated and then dissociated from the sensors to calculate *K*_D_. Data analysis used Octet Data Analysis HT 11.1 software (Sartorius), with a 1:1 binding model (a Fab has a single binding site, and no cooperativity was expected).

### Neutralization assay

A cell-based virus neutralization assay was used to examine the neutralizing activity of mAb and serum against EV-A71 and CV-A16. Before the assay, the serum sample was heat-inactivated at 56°C for 30 min. The serum and mAb samples to be tested were serially diluted, and 50 μl of the antibody preparation was mixed with an equal volume of 100 TCID_50_ (median tissue culture infectious dose) of the virus in a 96-well cell culture plate. After incubating at 37°C for 2 hours, 100 μl of rhabdomyosarcoma cell suspension (8 × 10^4^ cells) was added to each well. After incubating at 37°C in a CO_2_ incubator for 4 to 5 days, cytopathic effect was observed using an inverted microscope. Each experiment was carried out in triplicate. The cell control and virus back titration were set up for each experiment. The neutralization titer was determined by the highest sample dilution that completely inhibited the cytopathic effect in triplicate wells of the confluent monolayer of rhabdomyosarcoma cells.

### Synergistic neutralization assay

To assess the synergistic effects of mAbs against EV-A71, a cell-based neutralization assay was used. Antibody cocktails were prepared in a matrix format, combining a fixed concentration of the canyon-binding antibody with increasing concentrations of either twofold or threefold axis-binding antibodies and vice versa. The neutralizing activity for each combination was determined by the complete inhibition of the cytopathic effect in the confluent monolayer of rhabdomyosarcoma cells. All experiments were performed in duplicate. The neutralization data were formatted and analyzed using the SynergyFinder web tool to calculate synergy scores (*62*).

### In vivo study

Animal experiments were performed in accordance with the protocol approved by the Institutional Animal Care and Use Committee at the Chang Gung University, Taiwan (protocol no. CGU107-295). Animals were maintained, and experiments were carried out in accordance with the *Guide for the Care and Use of Laboratory Animals*, the recommendations of the Institute for Laboratory Animal Research and Association for Assessment and Accreditation of Laboratory Animal Care International standards.

The human SCARB2-transgenic mice (C57BL/6 background) (*25*) were maintained under specific pathogen–free conditions, and the in vivo effect of neutralizing mAbs was examined in the human SCARB2-transgenic mice infection model for clinical EV-A71 strains, genotype C4 12-73 and genotype B5 13-50144 viruses (*13*).

Three-week-old mice were randomly divided into groups and infected intraperitoneally with a lethal dose of EV-A71 24 hours before or after an intraperitoneal administration of mAb at a dose of 10 mg/kg. Mice were weighed daily, and mice with 20% weight loss were humanely euthanized.

### Ethics approval statement

Work with human peripheral blood mononuclear cells, B cell–derived mAbs, and convalescent serum was approved by the ethics committee at the National Taiwan University Hospital (202401065RINA) and the Chang Gung Medical Foundation (101-1901A3). Written informed consent was obtained from all donors.
