# Supplementary material for: Structural and functional mapping of protective human monoclonal antibodies against enterovirus A71
Source: Sci Adv. 2026 Jun 5;12(23):eaee8217. doi: 10.1126/sciadv.aee8217 (PMC13240189; doi:10.1126/sciadv.aee8217)
Supplement: Supplementary file 1 — Figs. S1 to S11 Tables S1 to S9 [file sciadv.aee8217_sm.pdf]

Science Advances
AAAS

Supplementary Materials for

Structural and functional mapping of protective human monoclonal antibodies against enterovirus A71

Daming Zhou *et al.*

Corresponding author: Daming Zhou, daming.zhou@zju.edu.cn;
Kuan-Ying A. Huang, arthurhuang1726@ntu.edu.tw; David I. Stuart, dave.stuart@strubi.ox.ac.uk

Sci. Adv. **12**, eaee8217 (2026)
DOI: 10.1126/sciadv.aee8217

This PDF file includes:

Figs. S1 to S11
Tables S1 to S9

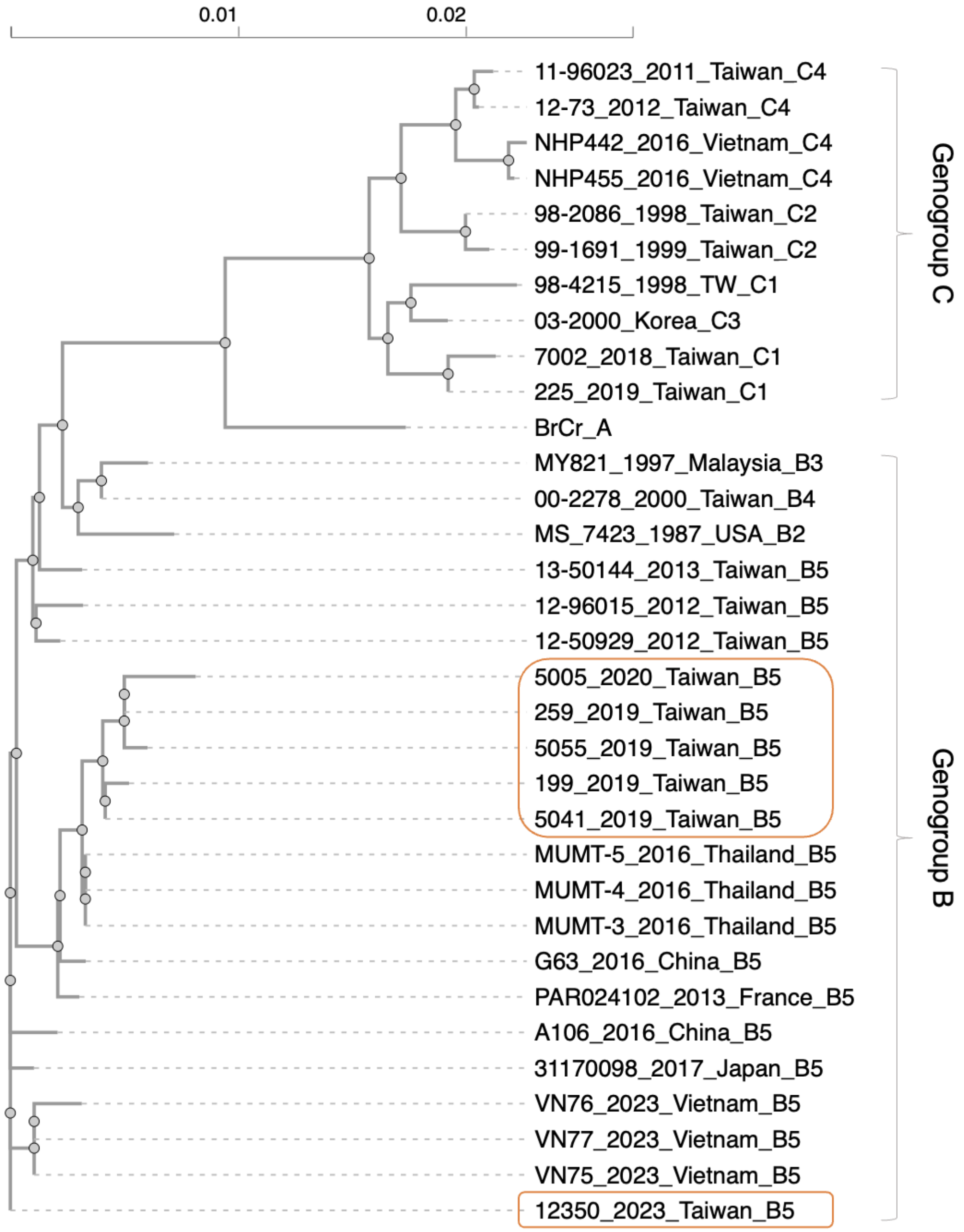

Figure S1. Phylogenetic tree. Based on VP2-VP3-VP1 amino acid sequences of the EV-A71 isolates identified in 2019-2020 and 2023 in Taiwan and other representative isolates.

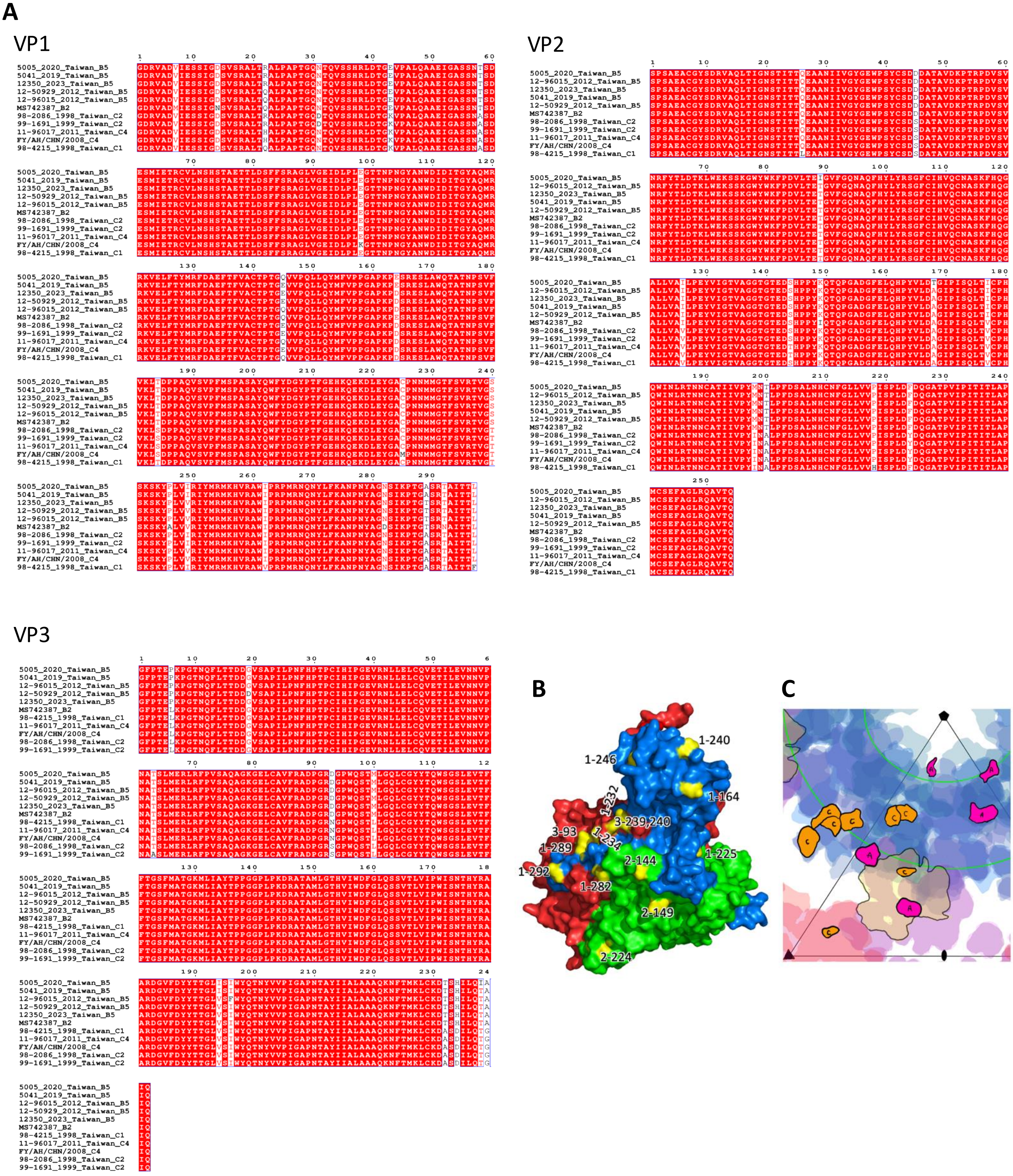

Figure S2. Sequence variation (**A**) Sequence alignment of subgenotypes used in the analyses of this paper. (**B**) Residue substitutions on the EV71 capsid. 1-, 2- and 3- represent VP1, VP2 and VP3 of EV71, respectively. (**C**) 10 amino acid substitutions among subtypes of EV-A71 mapping to the surface of the viral capsid.

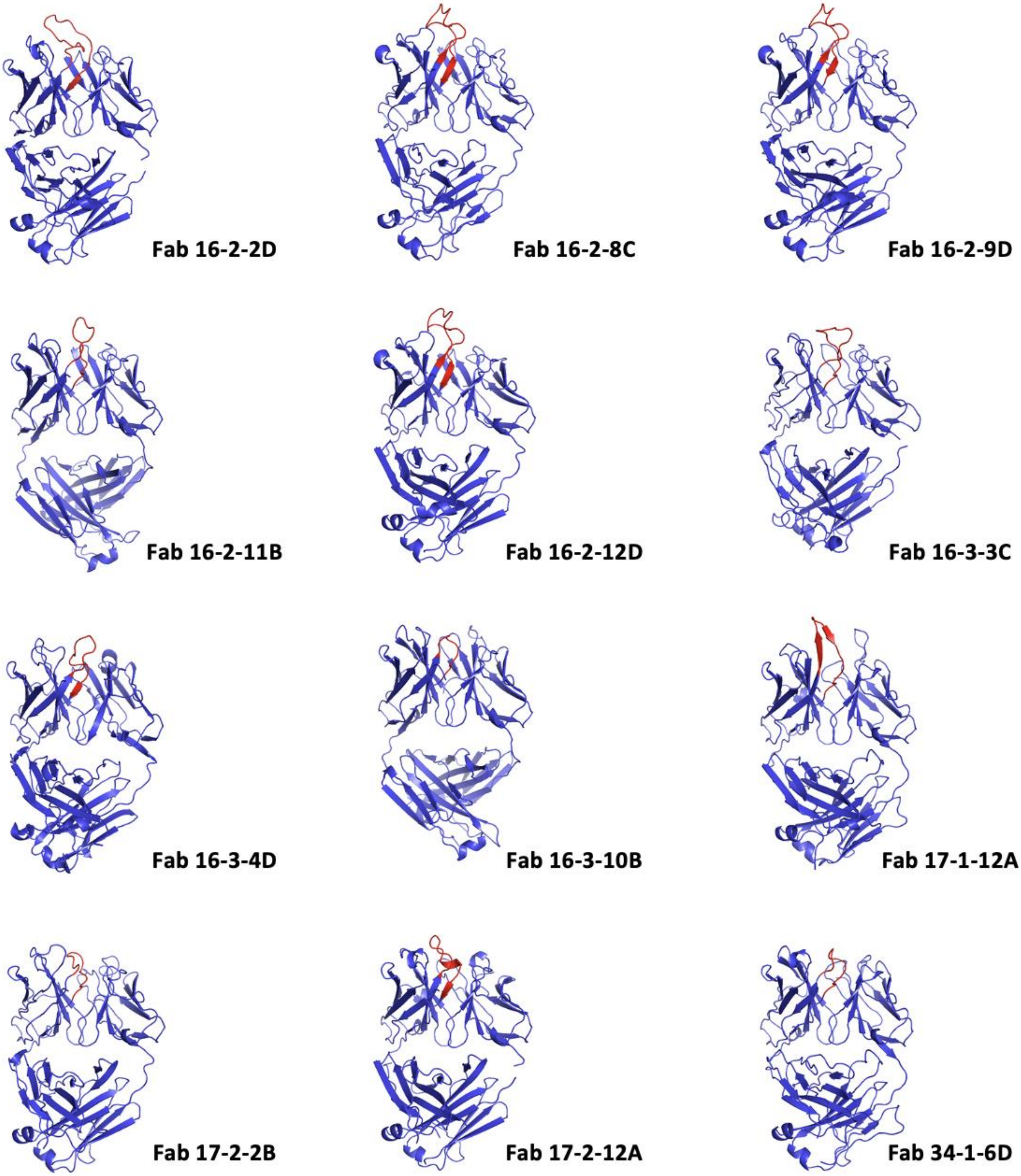

Figure S3. Crystal structures of anti-EV-A71 Fabs. The heavy chains are on the left and the CDR-H3s coloured red.

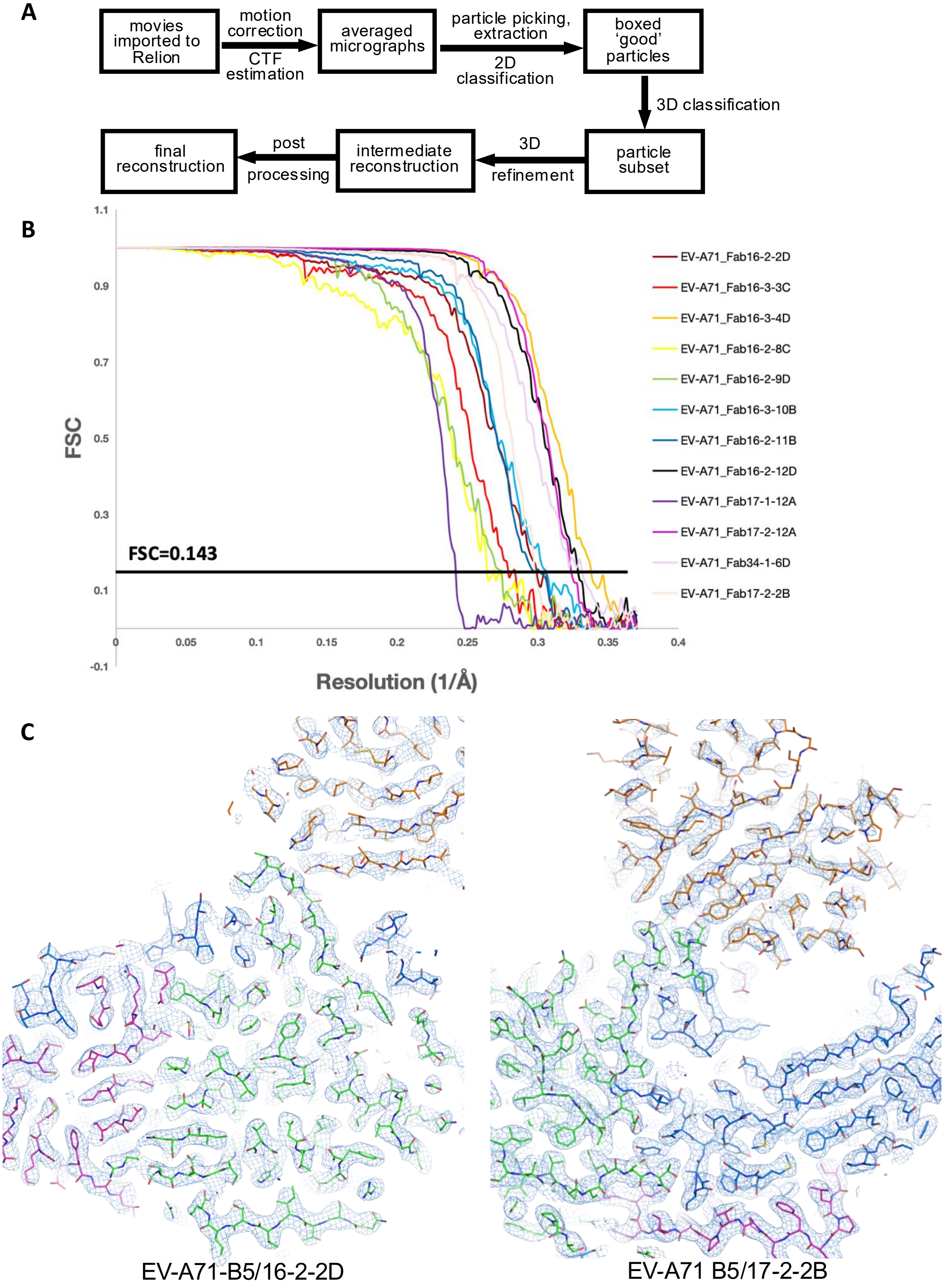

Figure S4. Cryo-EM structure determinations. (A) cryo-EM workflow. **(B)** The gold standard final map FSC curves of 12 EV-A71-Fab complexes at FSC = 0.143. **(C)** representative electron potential density for complexes EV-A71-B5/16-2-2D and EV-A71 B5/17-2-2B. VP1, VP2, VP3 and the Fab are coloured in blue, green, red and orange respectively.

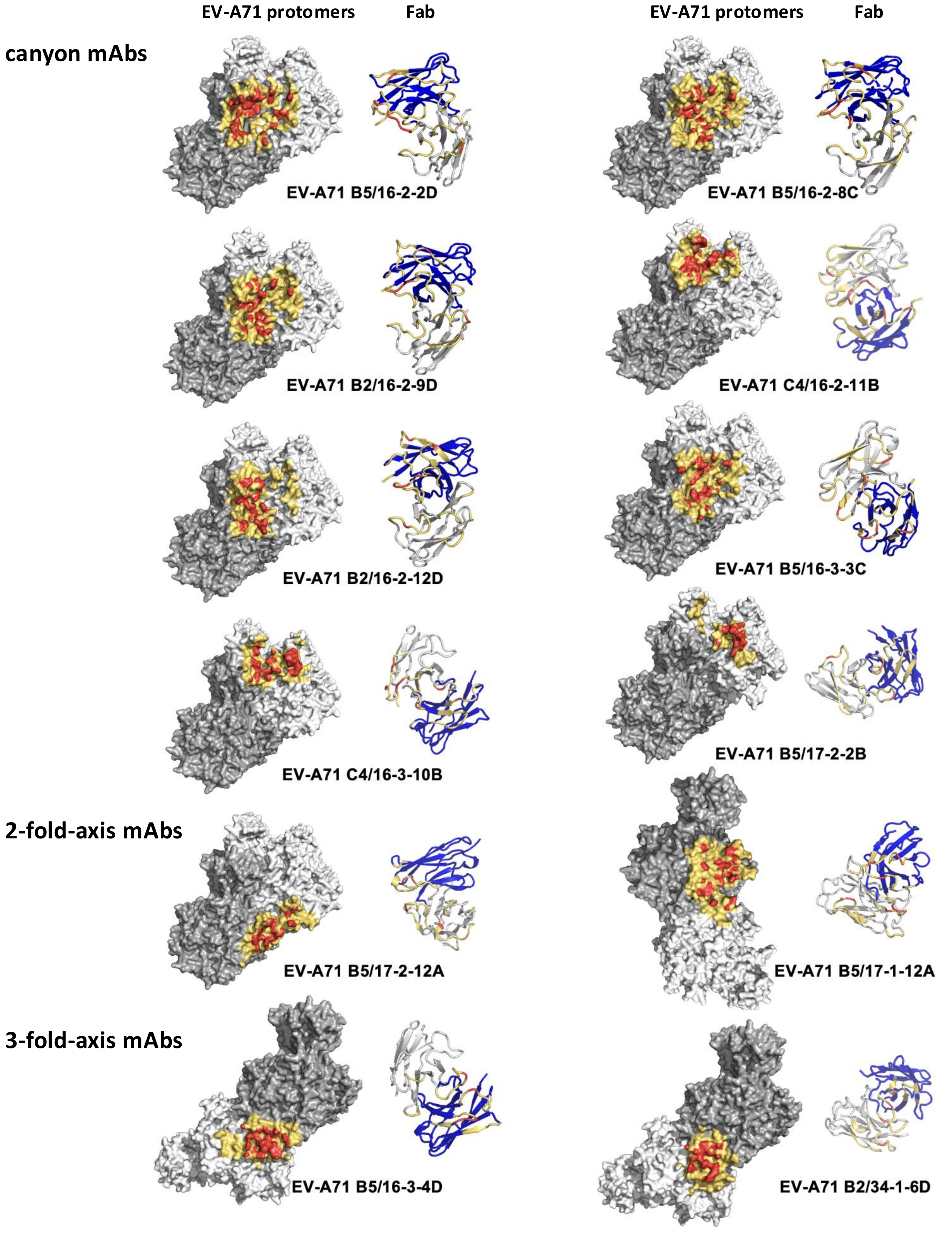

Figure S5. Surface and cartoon representations showing binding areas between two EV-A71 protomers and 12 different Fabs. Contact areas between virus and Fab are highlighted in red for distances ≤ 4.0 Å and in yellow for > 4.0 Å and ≤ 9.0 Å. The light and dark grey surfaces correspond to the two EV71 protomers. The heavy and light chain of Fabs are coloured in blue and grey, respectively.

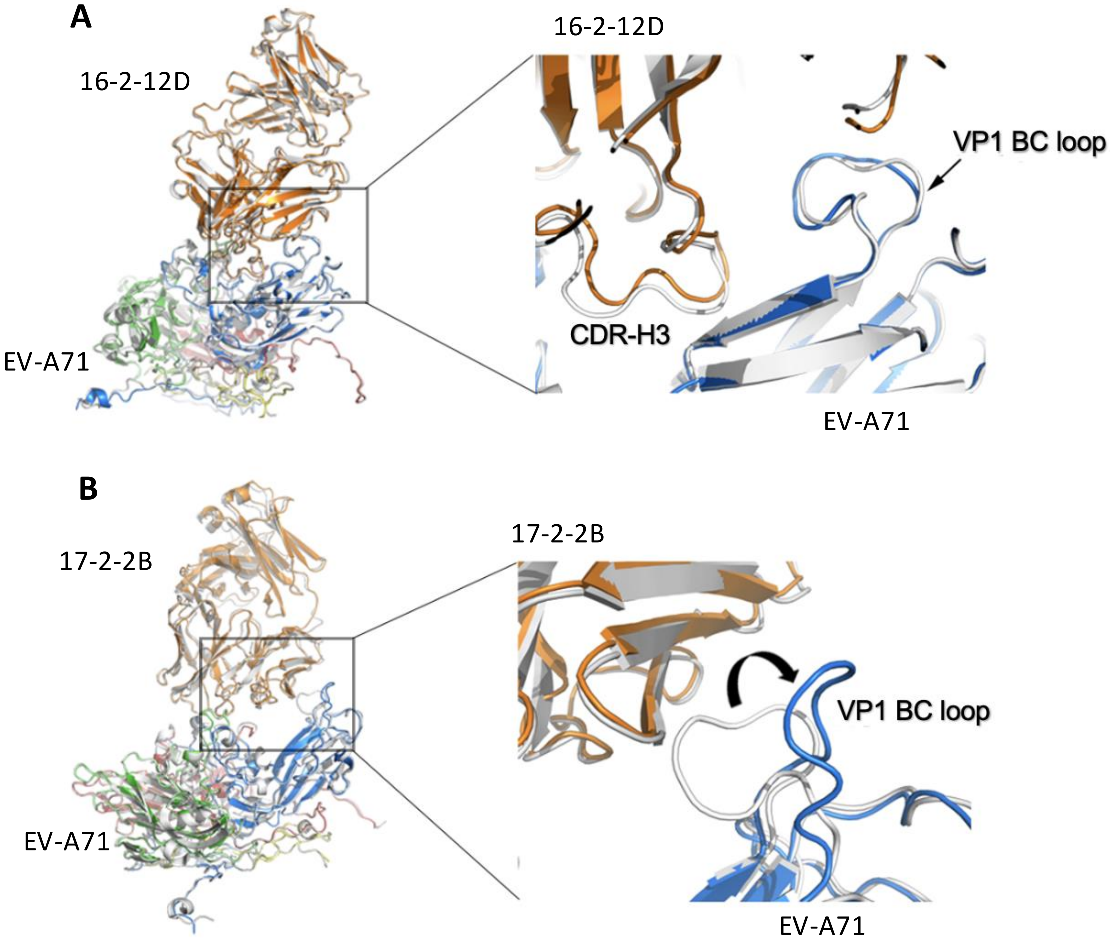

Figure S6. Conformational changes upon binding of 16-2-12D with EV-A71 B2 (A) and 17-2-2B with EV-A71 B5 (B). In the EV-A71/Fab complexes, VP1-4 of EV-A71 are coloured blue, green, red and yellow and Fabs are coloured in orange, respectively. The apo crystal structures of EV-A71 and Fabs are colored in grey.

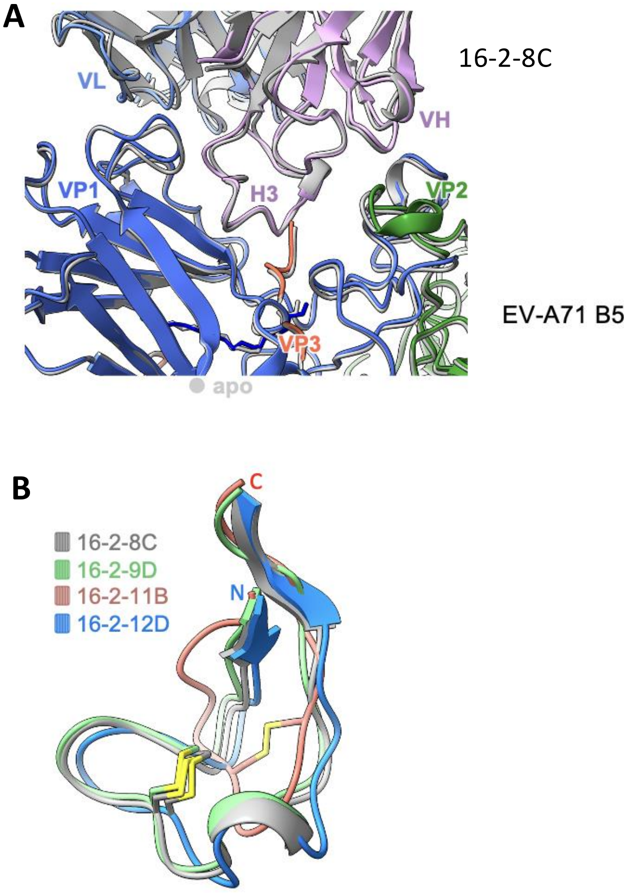

Figure S7. Conformational changes on binding. (**A**) Overlay of the apo structures (grey) of EV-A71 B5 and Fab 16-2-8C and their structure in the EV-A71-/Fab 16-2-8C complex (VH and VL of the Fab are coloured in light blue and pink, and VP1, 2 and 3 of EV-A71 are in dark blue, green and red). (**B**) Overlay of crystal structures of mAbs 16-2-8C, 16-2-9D, 16-2-11B and 16-2-12D CDR-H3. The disulphide bonds are shown in yellow.

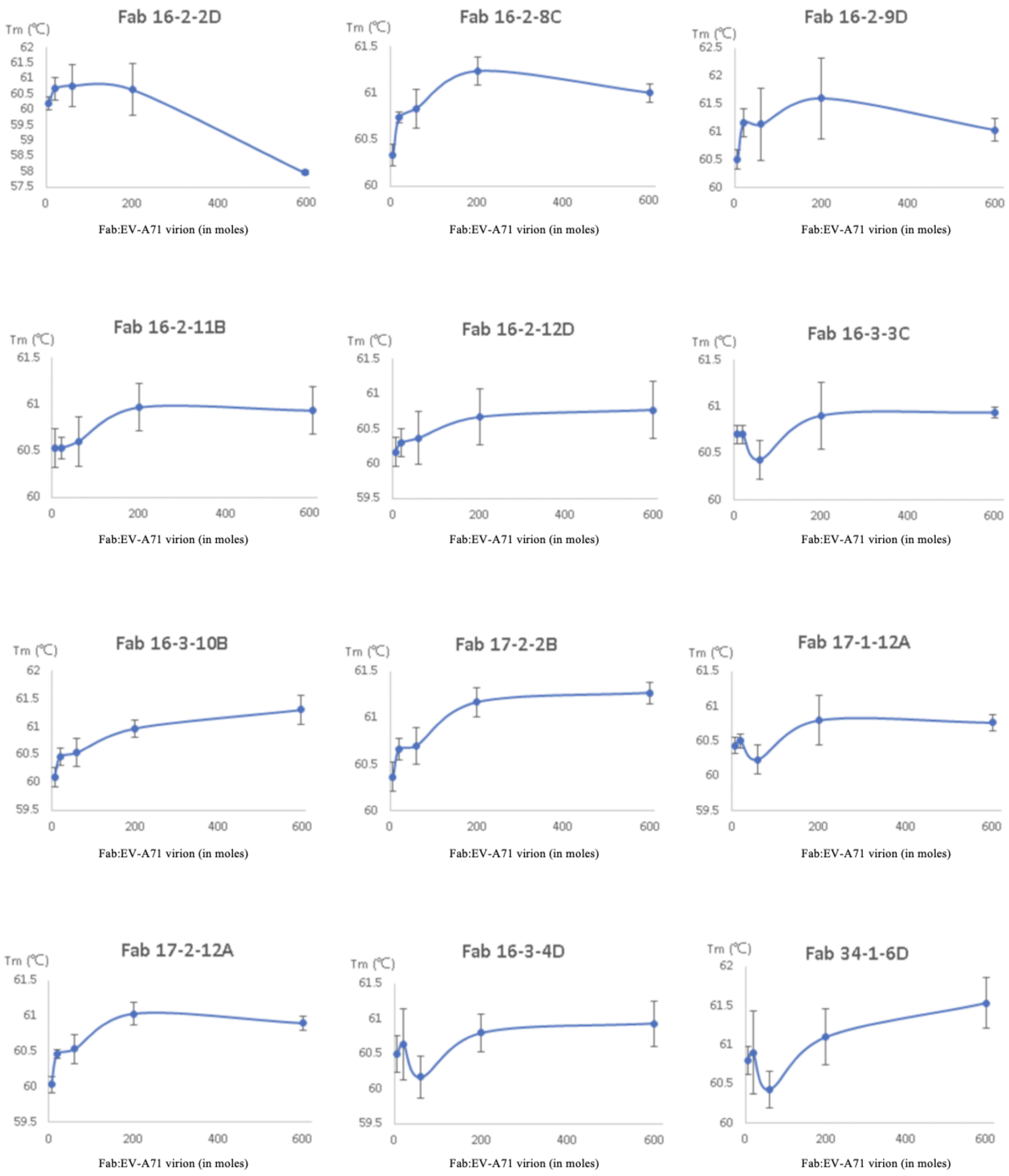

Figure S8. The effects of Fabs on the melting temperature of EV-A71 B5 virions (strain 12-96015) determined by thermal shift assay. The X axis represents the molar ration of Fab/EV-A71 capsid and Y the melting temperature.

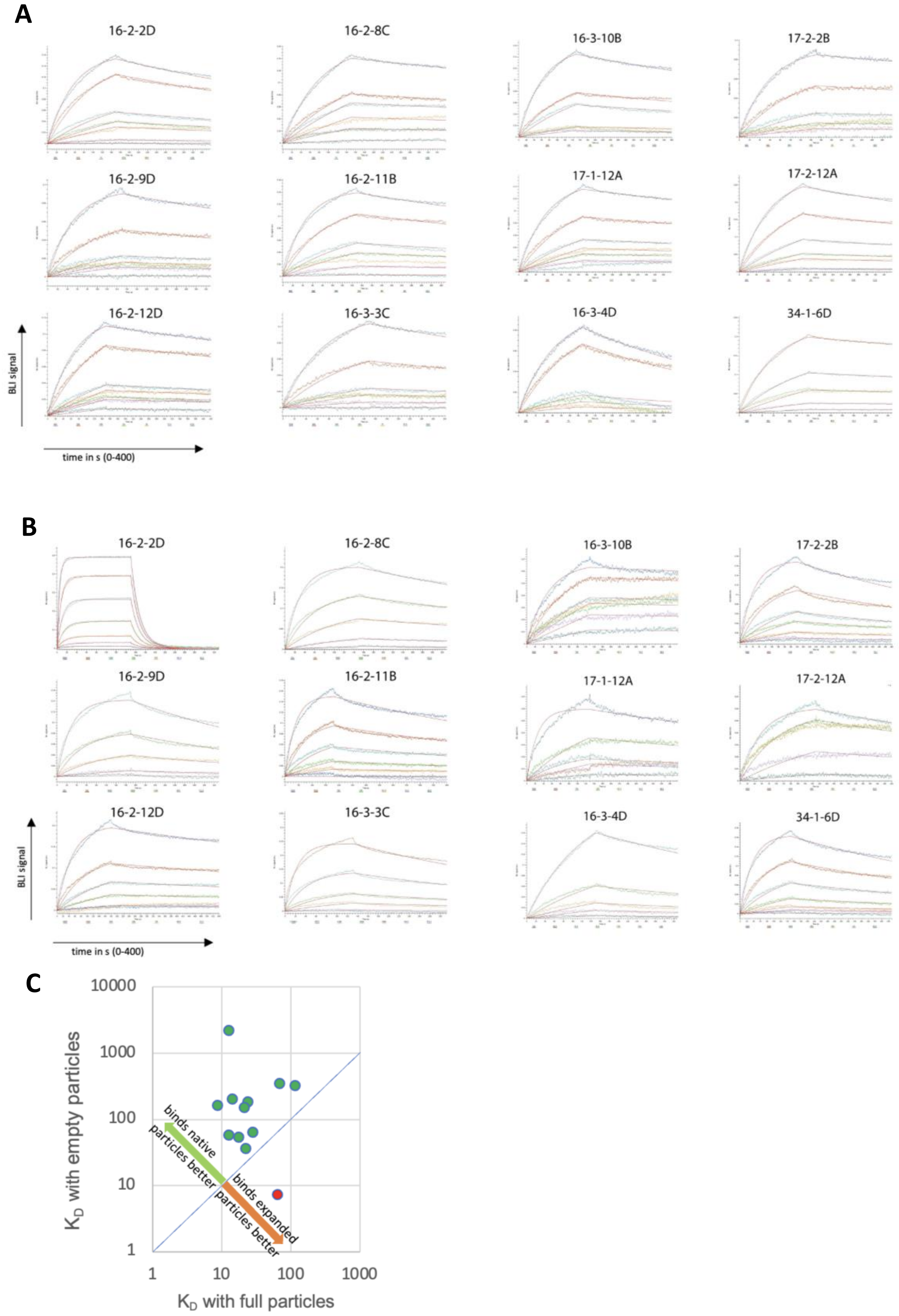

Figure S9. Binding affinities. Binding curves of Fabs with EV-A71 B5 (strain 12-96015) native particles (**A**) and expanded particles (**B**) measured by bio-layer interferometry. The different colours of the curves correspond to different concentrations (nM) of the Fabs used, prepared by 2-fold serial dilution. The red lines indicate the global model fit. Some imperfect curves were not used for the model fitting. (**C**) Scatter plot of binding KDs of Fabs with native and expanded particles. Most mAbs bind native particles better than expanded particles while 16-3-4D has a tighter binding with expanded particles than native particles.

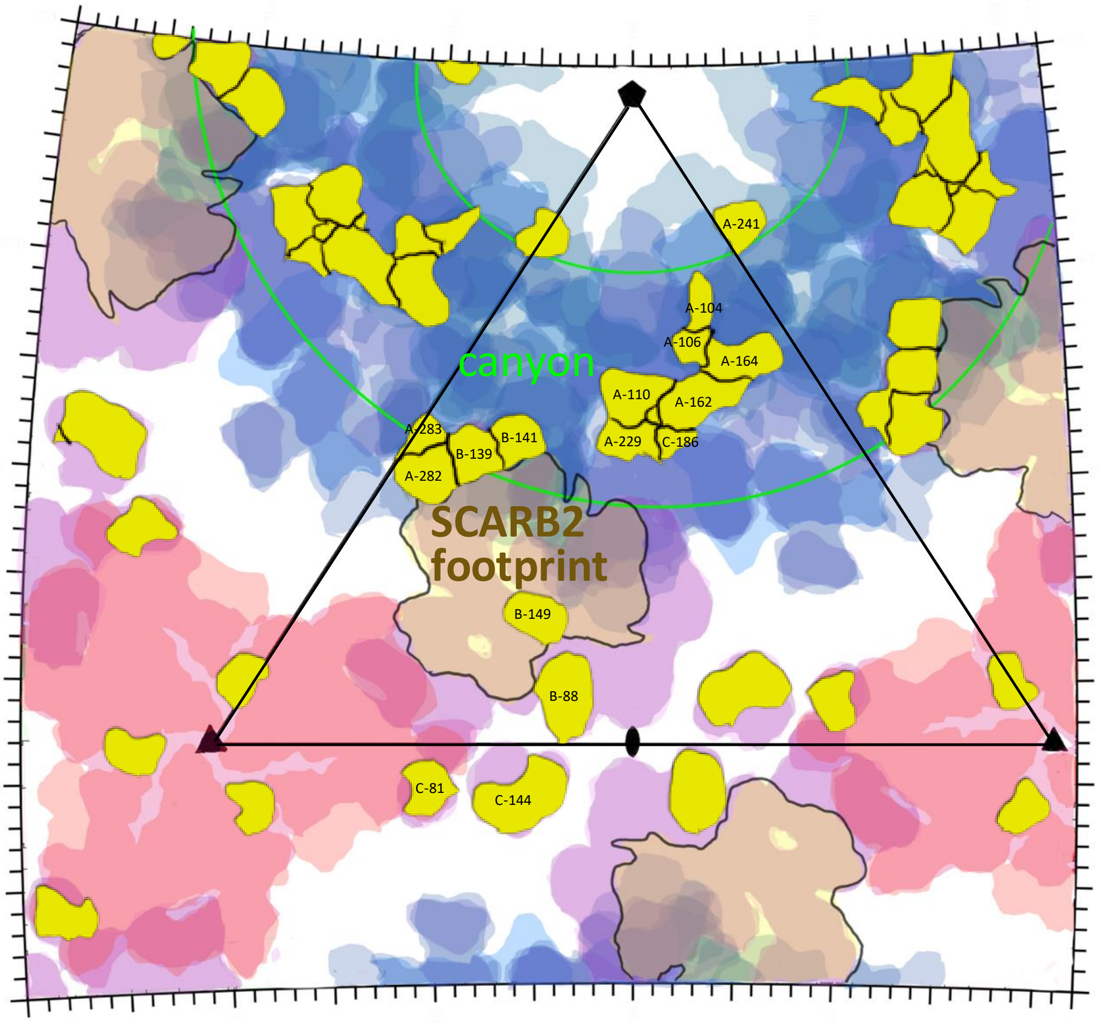

Figure S10. Roadmap showing sites of escape mutations (yellow, with labels A, B and C represents VP1, VP2 and VP3, respectively) on the EV-A71 capsid. Footprints of canyon binders are coloured in blue whilst 2- and 3-fold-axis binders are coloured purple and red respectively and the footprint of the SCARB2 receptor is drawn in light brown. Footprints are drawn with some transparency, so that the colours are more intense where they overlap. The canyon region is marked with two green lines.

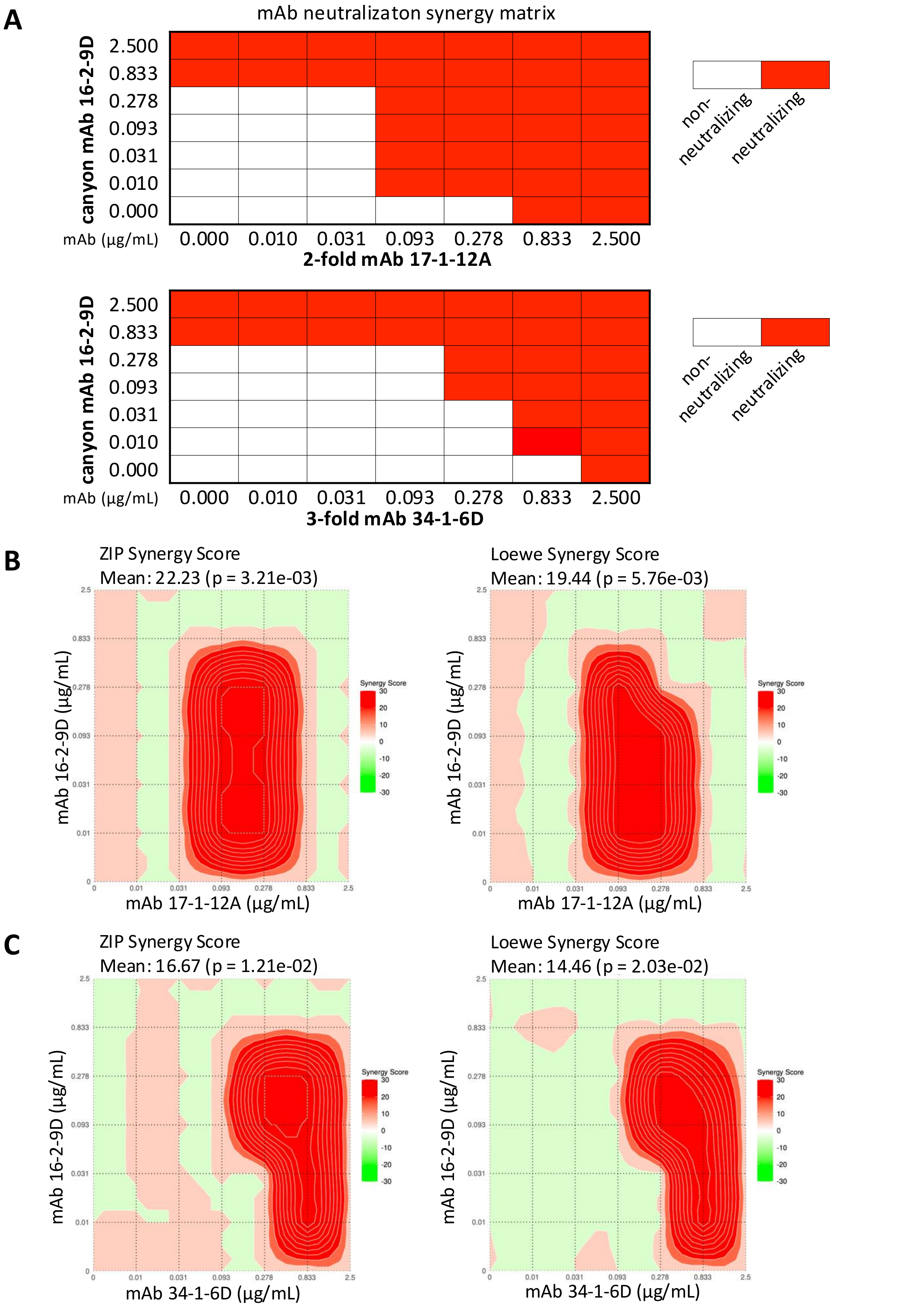

Figure S11. **Synergistic activity of of anti-EV-A71 monoclonal antibodies.** (**A**) Cross-neutralization dose-response matrices of antibody cocktails against genotype B5 EV-A71 (strain 12-96015). Upper panel cocktails consist of canyon-binding 16-2-9D and 2-fold binder 17-1-12A; lower panel shows 16-2-9D with 3-fold binder 34-1-6D. Neutralizing activity was assessed by cytopathic effect (non-neutralizing white, neutralizing red). Synergistic neutralization effects of (**B**) 16-2-9D and 17-1-12A and (**C**) 16-2-9D and 34-1-6D were analyzed using the web tool SynergyFinder. ZIP (Zero Interaction Potency) and Loewe synergy scores are shown. A synergy score greater than 10 indicates a likely synergistic interaction between the two antibodies (https://synergyfinder.aittokallio.group/synfin_docs/). Scores between -10 and 10 suggest an additive effect, while scores less than -10 indicate a likely antagonistic interaction. Both combinations show synergistic neutralization of EV-A71 *in vitro*.

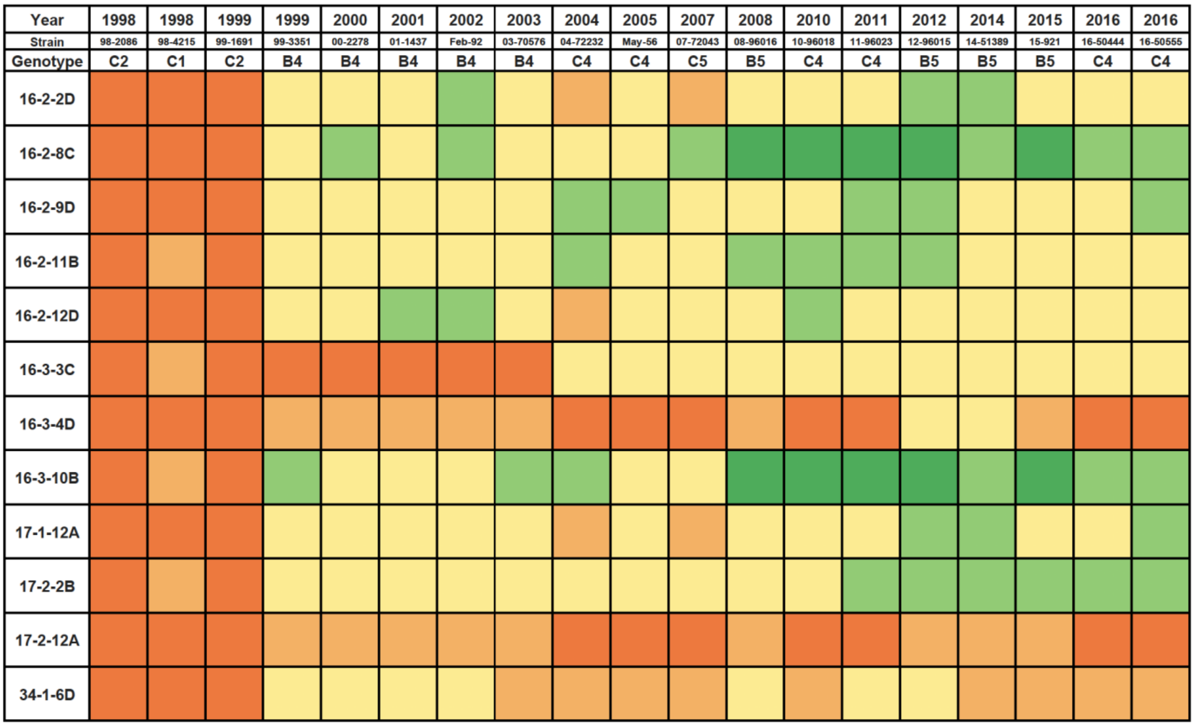

Table S1. Neutralization potency and breadth of 12 antibodies against different EV-A71 strains. Concentrations at which 100% neutralization was achieved: green, <100 ng/ml; pale green, 0.1–1 µg/ml; yellow, 1–10 µg/ml; orange, 10–50 µg/ml; red, no neutralization.

PDB/ID	Fab 16-2-2D 9I42	Fab 16-2-8C 9I3W	Fab 16-2-9D 9I43	Fab 16-2-11B 9I3X	Fab 16-2-12D 9I3Z	Fab 16-3-3C 9I41	Fab 16-3-4D 9I40	Fab 16-3-10B 9I41	Fab 17-1-12A 9I4C	Fab 17-2-2B 9I4D	Fab 17-2-12A 9I4B	Fab 34-1-6D 9I4E
**Data collection**												
wavelength (Å)	0.9763	0.9282	0.9282	0.9282	0.9282	0.9763	0.9282	0.9282	0.9282	0.9795	0.9686	0.9282
Space group	$P2_1$	$P2_1$	$P2_1$	$P2_1$	$C222$	$C222_1$	$P6_522$	$P\ 2_1$	$P622$	$P2_1$	$P1$	$P1$
Cell dimensions												
a, b, c (Å)	63.3, 104.0, 86.1	47.0, 80.4, 57.6	47.7, 80.3, 57.5	75.3, 83.9, 85.0	130.2, 265.3, 81.5	77.8, 156.6, 78.5	121.4, 121.4, 361.7	67.8, 50.1, 74.5	152.8, 152.8, 120.5	72.5, 71.7, 93.1	40.8, 72.6, 87.8	54.2, 56.0, 75.4
α, β, γ (°)	90, 104.0, 90	90, 93.8, 90	90, 94.9, 90	90, 103.5, 90	90, 90, 90	90, 90, 90	90, 90, 120	90, 116.5, 90	90, 90, 120	90, 105.8, 90	104.4, 93.2, 96.6	86.7, 86.4, 67.1
Resolution (Å)	1.92	1.14	1.18	2.57	3.06	1.32	2.78	2.12	2.83	2.56	2.68	1.63
No. reflections	82492/4093	153940/7168	141520/7009	32644/1595	27241/1299	112322/5448	40825/1957	25701/1197	20091/994	29400/1109	27165/1277	98557/4808
CC 1/2	1.0 (0.7)	1.0 (0.5)	1.0 (0.7)	1.0 (0.6)	1.0 (0.9)	1.0 (0.6)	1.0 (0.5)	1.0 (0.5)	0.98 (0.56)	1.0 (0.5)	1.0 (0.5)	1.0 (0.7)
I / σI	20.4 (1.3)	11.5 (1.5)	10.6 (2.0)	14.4 (1.6)	8.1 (1.4)	18.9 (1.4)	15.5 (1.1)	9.0 (1.5)	7.6 (1.2)	9.7 (1.0)	4.2 (1.2)	11.2 (1.5)
R_{merge}	0.14 (-)	0.05 (-)	0.07 (-)	0.06 (-)	0.5 (-)	0.1 (-)	0.1 (-)	0.1 (-)	0.3 (-)	0.2 (-)	0.3 (-)	0.04 (-)
Completeness	100 (100)	99.1 (92.6)	100 (99.6)	99.0 (97.7)	99.9 (97.2)	99.9 (98.2)	100 (97.0)	99.7 (92.8)	98.7 (99.5)	98.6 (74.5)	99.5 (90.8)	97.1 (94.6)
Redundancy	43.2 (8.4)	6.0 (4.2)	6.3 (4.9)	6.7 (5.9)	26.0 (27.0)	69.4 (29.4)	18.9 (18.5)	6.3 (5.1)	9.8 (10.0)	11.9 (3.0)	5.1 (5.2)	3.4 (2.8)
**Refinement**												
Resolution (Å)	44–1.92	47–1.14	57–1.18	87–2.57	21–3.06	78–1.32	91–2.78	61–2.12	76–2.83	50–2.56	48–2.68	72–1.63
No. reflections	78345/4018	137431/7459	133492/7025	30858/1588	25593/1413	99758/5587	37321/1957	23268/1229	19057/1026	27975/1397	25781/1313	88906/4593
No. mols / a.u.	2	1	1	2	2	1	2	1	1	2	2	2
R_{work} / R_{free}	0.164/0.192	0.166/0.194	0.145/0.177	0.209/0.255	0.210/0.255	0.143/0.174	0.241/0.277	0.204/0.236	0.223/0.267	0.194/0.243	0.208/0.253	0.158/0.209
No. atoms												
Protein	6724	3505	3445	6623	6630	3430	6656	3243	3400	6505	6507	6633
Ligand/ion	98	0	20	47	110	45	121	22	84	35	12	32
Water	419	565	606	87	0	507	14	177	19	107	111	674
B-factor												
Protein	50	20	19	79	64	21	92	51	60	51	53	39
Ligand/ion	68	-	27	71	104	42	147	71	103	77	49	37
Water	50	35	37	64	-	36	70	48	47	45	43	43
R.m.s. deviations												
Bond length (Å)	0.008	0.012	0.012	0.004	0.002	0.011	0.002	0.002	0.003	0.002	0.003	0.007
Bond angles (°)	1.0	1.6	1.8	0.7	0.6	1.7	0.6	0.5	0.6	0.6	0.6	1.4
Ramachandran												
Favored (%)	98.1	97.52	97.5	95.8	95.7	97.5	95.7	95.81	96.8	97.5	96.0	97.2
Allowed (%)	1.9	2.2	2.3	4.1	4.0	2.5	4.3	3.95	2.9	2.3	4.0	2.6
Outliers (%)	0	0.2	0.2	0.1	0.3	0	0	0.23	0.2	0.2	0	0.2

Table S2. X-ray Data collection and refinement statistics of Fabs (outer shell in parenthesis).

	EV-A71-B5/16-2-2D	EV-A71-B5/16-2-8C	EV-A71-B2/16-2-9D	EV-A71-C4/16-2-11B	EV-A71-B2/16-2-12D	EV-A71-B5/16-3-3C	EV-A71-B5/16-3-4D	EV-A71-C4/16-3-10B	EV-A71-B5/17-1-12A	EV-A71-B5/17-2-2B	EV-A71-B5/17-2-12A	EV-A71-B2/34-1-6D	Empty EV-A71-B5/16-3-3C
**PDB/ID**	**9RIK**	**9RIJ**	**9RIG**	**9RIQ**	**9RIH**	**9RIL**	**9RIM**	**9RIR**	**9RIN**	**9RIO**	**9RIP**	**9RII**	**9T6Z**
**Data collection and reconstruction**													
Voltage (kV)	300	300	300	300	300	300	300	300	300	300	300	300	300
Frames/movie	25	30	40	24	40	40	40	24	25	25	40	24	40
Dose rate (e^-/ Å^2/ s)	6	4	4	4	4	4	4	4	6	5	4	4	4
Total dose (e^-/ Å^2)	29	22	30	27	30	30	30	27	29	25	30	27	30
Pixel size (Å)	1.36	1.35	1.35	1.39	1.35	1.35	1.35	1.39	1.37	1.36	1.36	1.39	1.35
Defocus (µm)	0.5-2.5	0.5-2.5	0.5-2.5	0.5-2.5	0.5-2.5	0.5-2.5	0.5-2.5	0.5-2.5	0.5-2.5	0.5-2.5	0.5-2.5	0.5-2.5	0.5-2.5
Movies	903	424	2086	1129	468	1559	1177	1157	470	668	1170	1133	1559
Particles	1399	3665	3668	5881	16774	2770	26427	2852	3919	10037	40169	9700	823
Map resolution (Å)	3.4	3.8	3.7	3.3	3.1	3.6	3.0	3.3	4.1	3.4	3.1	3.0	4.8
Map sharpening B-factor (Å^2)	-103	-124	-145	-140	-133	-125	-128	-111	-175	-157	-151	-113	-137
**Model refinement**													
Model-to-map fit, CC_mask	0.88	0.68	0.66	0.85	0.78	0.81	0.77	0.84	0.84	0.87	0.85	0.79	0.37
R.m.s.d., bonds (Å)	0.004	0.003	0.007	0.003	0.002	0.003	0.003	0.004	0.002	0.003	0.003	0.002	0.006
R.m.s.d., angles (°)	0.6	0.5	0.9	0.5	0.5	0.5	0.5	0.5	0.5	0.5	0.5	0.5	0.9
All-atom Clash score	5.4	6.1	5.4	6.0	6.7	6.3	4.3	5.4	6.9	6.3	6.5	4.8	15.6
Rotamer outliers (%)	1.5	1.5	0.4	1.0	1.2	1.9	1.4	0.9	1.1	1.0	1.2	1.2	6.4
**Ramachandran plot**													
Favored (%)	96.0	95.9	95.4	94.8	97.4	95.9	95.5	96.3	95.9	96.2	94.8	96.4	88.9
Allowed (%)	4.0	4.1	4.6	5.2	2.6	4.0	4.5	3.7	4.1	3.8	5.2	3.6	8.1
Outliers (%)	0	0	0	0	0	0.1	0	0	0	0	0	0	3.0

Table S3. Cryo-EM data collection and refinement statistics of EV-A71-Fab complexes.

Fab	Buried solvent accessible area (Å^2)								
	HC-VP1	HC-VP2	HC-VP3	HC-EV71	LC-VP1	LC-VP2	LC-VP3	LC-EV71	Fab-EV71
**Canyon binders**									
16-2-2D	785	144	165	1094	329	0	257	586	1680
16-2-8C	756	121	232	1109	473	0	144	617	1726
16-2-9D	731	133	230	1094	406	0	125	531	1625
16-2-11B	546	176	233	955	463	0	0	463	1418
16-2-12D	711	173	166	1050	279	0	24	303	1353
16-3-3C	610	16	293	919	310	75	8	393	1312
16-3-10B	174	318	14	505	530	83	287	900	1405
17-2-2B	234	151	106	491	222	160	102	483	974
**2-fold-axis binders**									
17-1-12A	231	720	218	1169	161	153	0	315	1484
17-2-12A	235	617	35	887	110	46	230	385	1272
**3-fold-axis binders**									
16-3-4D	0	642	297	939	0	27	20	47	986
34-1-6D	0	492	120	612	0	59	259	317	929

Table S4. Solvent accessible areas between chains of Fabs (HC: heavy chain; LC: light chain) and viral proteins of EV-A71 in the EM structures of 12 EV-A71-Fab complexes.

Complex	Fab	Virus
EV-A71-B5/16-2-2D	CDR-H3, CDR-H2	VP1 B-C loop
EV-A71-B5/16-2-8C	none	none
EV-A71-B2/16-2-9D	none	none
EV-A71-C4/16-2-11B	CDR-H3	VP1 B-C loop
EV-A71-B2/16-2-12D	CDR-H2, CDR-H3	none
EV-A71-B5/16-3-3C	CDR-L2, CDR-H3	none
EV-A71-C4/16-3-10B	CDR-L2	none
EV-A71-B5/17-2-2B	none	VP1 B-C loop
EV-A71-B5/17-1-12A	none	VP1 GH loop, C terminus of VP2
EV-A71-B5/17-2-12A	CDR-H3	VP1 GH loop
EV-A71-B5/16-3-4D	CDR-H2	none
EV-A71-B2/34-1-6D	CDR-H3	none

Table S5. Regions showing significant conformational changes of Fab and EV-A71 upon complex formation.

	Heavy chain			Light chain	
**Antibody**	**Top V gene match**	**Top D gene match**	**Top J gene match**	**Top V gene match**	**Top J gene match**
16-2-2D	IGHV1-46*01/ IGHV1-46*02	IGHD3-22*01	IGHJ6*02	IGLV1-44*01	IGLJ1*01
16-2-8C	IGHV4-39*03	IGHD2-2*01	IGHJ4*02	IGLV1-44*01	IGLJ2*01/IGLJ3*01
16-2-9D	IGHV4-39*01	IGHD2-2*01	IGHJ4*02	IGLV1-44*01	IGLJ2*01/IGLJ3*01
16-2-11B	IGHV1-46*01	IGHD2-21*02	IGHJ4*02	IGLV2-14*01	IGLJ2*01/IGLJ3*01/ IGLJ3*02
16-2-12D	IGHV4-39*01	IGHD2-2*01	IGHJ4*02	IGLV1-47*02	IGLJ2*01/IGLJ3*01
16-3-3C	IGHV3-9*01	IGHD6-19*01	IGHJ6*02	IGLV2-11*01	IGLJ2*01/IGLJ3*01/ IGLJ3*02
16-3-10B	IGHV7-4-1*02	IGHD1-14*01	IGHJ5*02	IGLV2-23*01/ IGLV2-23*03	IGLJ1*01
17-2-2B	IGHV4-39*01	IGHD3-10*01	IGHJ4*02	IGLV2-8*01	IGLJ3*02
17-1-12A	IGHV3-11*05	IGHD1-26*01	IGHJ6*02	IGKV2-28*01/ IGKV2D-28*01	IGKJ2*02
17-2-12A	IGHV3-23*04	IGHD3-16*01	IGHJ6*02	IGLV7-43*01	IGLJ3*02
16-3-4D	IGHV4-39*01	IGHD6-19*01	IGHJ6*02	IGLV1-44*01	IGLJ3*02
34-1-6D	IGHV7-4-1*02	IGHD1-7*01	IGHJ6*02	IGKV1-39*01/ IGKV1D-39*01	IGKJ4*01

Table S6. Predicted heavy and light chain gene usage of 12 anti-EV-A71 antibodies.

	Sequence of CDR-H3	Length of CDR-H3 (aa)
**Canyon binders**		
16-2-2D	GPGPGGKYYYDSSDAYYYYGMDV	23
16-2-8C	HSSPQ**C**SPTS**C**YEGPYTRDWYVDY	24
16-2-9D	HSSPQ**C**SPTS**C**YEGPYTRDWYVDY	24
16-2-11B	NYNGY**C**AGD**C**YSPDF	15
16-2-12D	HASPH**C**SSTS**C**YDGPYNKNWYVDL	24
16-3-3C	DGPSSGWSYQNYYNAMDV	18
16-3-10B	DPLGNWFDP	9
17-2-2B	TYGSGSYWGYFEY	13
**2-fold-axis binders**		
17-1-12A	EKWEKLGKLYYYGLDV	16
17-2-12A	SVAARRFYFYYGMDA	15
**3-fold-axis binders**		
16-3-4D	HVPVAGFGYYYYGMDV	16
34-1-6D	AKALLYYGMDV	11

Table S7. Sequences and lengths of CDR-H3s of the anti-EV-A71 antibodies. Canyon binders (except antibodies 16-3-10B and 17-2-2B) tend to have longer CDR-H3s than 2- and 3-fold-axis binders. Cysteine residues in bold form a disulphide bond in the corresponding antibody.

mAb	pre-attachment EC_{50}(μg/ml)	post-attachment EC_{50} (μg/ml)	Binding K_Ds with EV71 virion (nM)	Binding K_Ds with EV71 empty particle (nM)	neutralizing ability	overlapped volume Fab_VHVL with SCARB2 (Å^3)
16-2-2D	0.27	38.53	12.6 ± 0.11	2207 ± 10.5	+++	412 (+)
16-2-8C	0.27	109.78	14.1 ± 0.19	204.6 ± 1.55	+++	339 (+)
16-2-9D	1.41	99.96	68.4 ± 0.92	349.6 ± 2.8	+++	355 (+)
16-2-11B	0.28	7.55	27.8 ± 0.43	63.2 ± 0.53	++++	0 (-)
16-2-12D	1.5	>400	17.7 ± 0.15	53.5 ± 0.39	++	421 (+)
16-3-3C	0.28	7.61	117 ± 1.85	320 ± 3.39	++++	1207 (+++)
16-3-10B	0.05	1.41	22.8 ± 0.19	36.3 ± 1.44	+++++	306 (+)
17-2-2B	0.05	1.39	24 ± 0.66	184.4 ± 1.35	+++++	631 (++)
17-1-12A	1.42	58.83	8.6 ± 0.09	161 ± 2.16	++	6643 (+++++)
17-2-12A	234.24	292.77	12.7 ± 0.08	58 ± 0.81	+	2907 (++++)
16-3-4D	4.37	>400	66.3 ± 0.92	7.2 ± 0.11	++	2960 (++++)
34-1-6D	1.43	267.15	21.4 ± 0.29	148.6 ± 0.99	++	1605 (++++)

Table S8. Pre- and post-attachment neutralizing abilities of anti-EV-A71 mAbs. Binding KDs of 12 anti-EV-A71 Fabs with EV-A71 full or empty particles and overlapped volume of mAbs' VHVL parts with SCARB2 (for ease of comparison the neutralizing ability and volume are mapped onto a simple – to +++++ scale). Virus used for all experiments here is EV-A71 genotype B5 (strain 12-96015).

mAb	EV-A71 B5 (strain 12-96015)	EV-A71 C4 (strain 11-96023)
16-2-2D	VP1 Y106H	VP1 S241F
16-2-8C	VP1 T232A	VP1 T232A
	VP1 M229I	
16-2-9D	VP1 D110G	VP1 T232A
	VP1 T232A	VP1 K162E
	VP1 M229K	
16-2-11B	VP1 N104K	VP1 S241F
16-2-12D	VP3 F186L	VP1 D110G
	VP1 T232A	
16-3-3C	VP1 D164G	VP1 D164N
		VP1 D164V
16-3-10B	VP1 N282D	VP2 T139I
		VP2 T141M + VP1 S283F
17-2-2B	VP2 T141I	VP2 T141M
	VP1 S283F	
17-1-12A	VP2 K149E	VP2 E88V
	VP2 K149G	VP2 K149E
17-2-12A	VP3 K144E	Not done
	VP2 K149E	
	VP3 K144E + VP3 T148A	
16-3-4D	VP3 E81G	Not done
34-1-6D	VP3 E81G	VP3 E81K
	VP2 D225N	

Table S9. Substitutions of amino acids on the EV-A71 capsid causing escape mutants of EV-A71 strains 12-96015 (B5) and 11-96023 (C4) from anti-EV-A71 human mAbs.
